# Global meta-analysis of drought associated rice rhizosphere microbiome data and soil application of selected bacteria reveals potential of *Pantoea dispersa* for drought stress alleviation in rice plants

**DOI:** 10.3389/fpls.2026.1904089

**Published:** 2026-09-03

**Authors:** Indrani Sharma, Ramen Barman, Sampurna Kashyap, Arnav Patowary, Bhaskar Jyoti Parasar, Poonam Jyoti, Niraj Agarwala

**Affiliations:** 1 Department of Botany, Gauhati University, Gopinath Bordoloi Nagar, Jalukbari, Guwahati, Assam, India; 2 Department of Botany, Dhing College, Dhing, Nagaon, Assam, India; 3 Department of Biotechnology, Council of Scientific & Industrial Research - Institute of Himalayan Bioresource Technology, Palampur, India

**Keywords:** drought, plant growth-promotion, rhizospheric bacteria, rice, susceptible, tolerant

## Abstract

**Introduction:**

Drought stress poses a significant threat to global rice productivity, jeopardizing crop yield and food security, particularly in water-limited agroecosystems. The application of plant-associated microbes to enhance drought resilience in crops has gained considerable attention in recent years. However, systematic investigation on changes in rice rhizosphere microbial diversity under drought stress remains limited. This study aimed to identify drought-responsive bacterial taxa and evaluate selected rice rhizosphere bacteria for their potential to enhance drought tolerance in rice.

**Methods:**

A meta-analysis of 16S rRNA amplicon sequencing datasets from rice drought-associated studies was conducted to identify drought-responsive bacterial taxa. Based on the findings of meta-analysis and previous literature, five pre-isolated rice rhizospheric bacterial strains belonging to the genera *Streptomyces, Burkholderia, Pantoea, Pseudomonas*, and *Variovorax* were selected and characterized for key plant growth-promoting traits and osmotic stress tolerance using 25% of PEG. The selected isolates were subsequently evaluated for their potential to enhance drought tolerance in two Indian rice varieties, CR Dhan 802 (drought-tolerant) and Ranjit (drought-susceptible) under pot conditions.

**Results:**

Based on meta-analysis of 16S amplicon datasets and literature review bacterial isolates possessing drought tolerance and plant growth-promoting traits, were selected for the pot experiments. Soil inoculation with selected bacterial strains showed that the treatment groups *Pantoea dispersa* NA-08, *Burkholderia cepacia* NA-07, and Consortium-I (*S. jiujiangensis* NA-06, *B. cepacia* NA-07 and *P. dispersa* NA-08) alleviated drought stress in both the rice varieties. Furthermore, these treatment groups showed significant enhancement in plant anti-oxidative and soil enzymatic activities. However, the treatments with *Streptomyces jiujiangensis* NA-06 and *Variovorax guangxiensis* NA-16 exhibited growth promotion in rice plants, but no significant alleviation of drought stress was observed.

**Conclusion:**

The isolate *P. dispersa* NA-08 showed the higher potential for enhancing rice drought tolerance in pot experiments and might serve as a promising isolate for evaluation under field conditions.

## 1 Introduction

Global food security faces a remarkable challenge due to the intensifying impacts of drought, one of the most destructive abiotic stresses in modern agriculture. Over the past decades, shifting precipitation patterns and rising temperatures have drastically increased the frequency and intensity of drought events, leading to the substantial crop yield losses across diverse ecological ranges (Niu et al., 2025). Drought stress significantly disturbs a cascade of plant physiological processes such as chlorophyll content, stomatal conductance, and osmotic balance leading to oxidative stress through reactive oxygen species (ROS) accumulation and disruption of biochemical homeostasis (Mahreen et al., 2023; Parasar et al., 2024; Sharma and Joshi, 2025). Notably rice (*Oryza sativa* L.), a high-water demanding crop plant, is facing a major challenge in drought stress conditions limiting its productivity (Bhandari et al., 2023). However, the degree of drought sensitivity varies considerably among rice varieties, with contrasting genotypes exhibiting marked differences in physiological and molecular responses under water deficit conditions (Melandri et al., 2021). Yet, the consequences of drought further destabilize the soil-dwelling microbial communities that perform essential ecosystem functions, including nutrient cycling and carbon (C) sequestration. Reduced soil moisture lowers substrate availability and exposes microbial cells to desiccation stress, collectively suppressing microbial activity and restructuring community composition (Manzoni et al., 2012). In response, soil microbiota utilizes a repertoire of adaptive strategies, which include production of osmolytes, higher investments in enzyme synthesis for substrate acquisition, or stress avoidance strategy, such as dormancy to survive and persist under water limited condition (Manzoni et al., 2014; Schimel, 2018). Climate change-induced disruption of these microbial dynamics threatens to weaken the ecological processes accounting for the global climate and soil health conditions (Metze et al., 2023).

Evidence based on multiple studies demonstrates that drought drives pronounced shifts in soil microbial community composition. Across diverse crop species and geographic regions, drought conditions selectively enrich members of Actinobacteria, Pseudomonadota, Firmicutes, Chloroflexi, Acidobacteriota, Gemmatimonadota, and Patescibacteria (Santos-Medellín et al., 2017; Xie et al., 2021; Metze et al., 2023; Wu et al., 2024; Abdelrahman et al., 2025; Zacamo-Velázquez et al., 2026). These recurrent patterns suggest possible plant-associated selection of drought-responsive microbial communities, although methodological and environmental differences among studies may also contribute. The plants and their associated microbiota forms a dynamic, mutually reinforcing relationship to navigate water stress (Metze et al., 2023; Liu et al., 2024; Li, J. et al., 2025). 

The rhizosphere, a region in root soil proximity, serves as a critical zone where plant-microbe interactions collectively contribute towards drought stress adaptation (de Vries et al., 2020; Muhammad et al., 2024). The improvement in plant responses to various stress conditions is largely mediated by the associated microbes, producing different metabolites, nutrients, phytohormones, and osmolytes that aid plants in maintaining cellular integrity under stressful circumstances (Benmrid et al., 2023; Sharma et al., 2023). Several bacterial genera belonging to the phyla Actinobacteriota, Pseudomonadota, and Firmicutes have been widely studied and used as bioinoculants for improving crop drought tolerance. Within Actinobacteria, the *Streptomyces* spp. have been widely reported as a potential candidate for promoting plant growth and tolerance to stress conditions (Liu et al., 2024; Pang et al., 2024; Li, J. et al., 2025; Maldonado et al., 2026). For example, *Streptomyces rimosus* C-2012 and *S. monomycini* C-801 results in significant enrichment in plant growth, biomass and essential oil yield of peppermint under water deficit condition (Esmaeil et al., 2019). Similarly, inoculation of tomato with *Streptomyces* sp. enhanced plant growth and anti-oxidative enzyme activities and suppresses the root specific stress responsive genes *ERF1* and *WRKY70* (Abbasi et al., 2020). Additionally, seed bio-priming of wheat with *Pseudomonas azotoformans* JRBHU5 and *Burkholderia seminalis* JRBHU6 results in drought stress tolerance (Prasad et al., 2025). Furthermore, co-inoculation of *Pantoea agglomerans* BCH-1 and *Bacillus pseudomycoides* BCH-3 in drought-sensitive and drought-tolerant maize cultivars significantly improved plant growth parameters and activated the defense system under water deficit conditions (Tariq et al., 2025). Similar beneficial effects of plant growth promoting bacteria such as *Bacillus* spp. have been reported in sugarcane, which significantly enhance plant drought tolerance (Buqori et al., 2025). Application of plant growth promoting bacteria such as *Paraburkholderia* and *Delftia* (Islam et al., 2023), *Bacillus megaterium* (Lee et al., 2024), *Bacillus pumilus* SH-9 (Shaffique et al., 2024), and *Micrococcus* sp. TCR-5 (Kashyap et al., 2026) significantly enhances drought stress tolerance in rice. Collectively, these findings underscore the ecological and agricultural significance of drought associated bacterial taxa that might aids in the development of targeted bioinoculant strategies for drought-prone agricultural crop systems. Such approaches offer sustainable alternatives of traditional drought management practices and contribute towards improved crop resilience under changing climatic scenarios. Therefore, the present study aimed to (i) conduct global meta-analysis of 16S rRNA amplicon datasets to identify drought-associated bacterial taxa in rice rhizosphere (ii) identify widely reported drought-responsive plant growth promoting bacteria across diverse crop plants from literature, and (iii) evaluate the drought-tolerance potential of representative rhizospheric bacterial isolates selected on the basis of both meta-analysis of 16S rRNA amplicon datasets and literature survey, through pot experiment using contrasting Indian rice varieties.

## 2 Materials and methods

### 2.1 Selection and analysis of drought-associated rice rhizosphere microbiome data sets

Initially, we conducted a literature survey across multiple publicly available databases, including Google Scholar and Web of Science (WoS), using the keywords ‘rice’ AND ‘drought’ AND ‘microbiome’. A total of fourteen studies comprising rice rhizosphere and bulk soil sample datasets were compiled for this meta-analysis. The raw 16S amplicon datasets were then downloaded from the selected studies using their accession numbers. A metadata file was maintained to store the relevant information of each dataset. Overall, we compiled 601 samples from bulk soil (291 control and 310 drought) and 482 samples from rice rhizosphere (216 control and 266 drought). Different sets of primers, such as 322F-796R, 338F-806R, 341F-805R, 343F-798R, 515F-806R, 799F-1193R, and 520F-799R were used to generate the amplicon data. All sample details with sequencing parameters, including primer sequences, and sequencing platforms are listed in **Supplementary Table 1**. The obtained raw sequence data from each study were processed individually using DADA2 pipeline (v1.28; Callahan et al., 2016) in R. Before the analysis, primer sequences were removed using Cutadapt (v4.4; Martin, 2011). The resulting files were further processed for quality filtering, denoising, dereplication and chimeric sequences removal. The obtained feature tables from all the studies were then merged for taxonomic aggregation using the SILVA (v138.1) database (Quast et al., 2013). For downstream analysis of our microbiome data, ‘microeco’ R package (Liu et al., 2021, 2026) was used. Low-abundance taxa were filtered using a relative abundance value ≥ 0.0001 and frequency of occurrence ≥ 2. Rarefaction curves were examined to confirm that the selected sequencing depths adequately captured bacterial diversity across samples. For the bulk soil datasets, samples were rarefied at 4000, retaining 456 out of the 601 samples. In the case of rhizosphere datasets, samples were rarefied to 4900 sequences, retaining 399 out of the 482 samples for further analyses. The relative abundance graphs were generated using the package ‘ggplot2’ (Wickham, 2016) in R. The differential abundance analysis was performed at the genus level using linear discriminant analysis Effect Size (LEfSe), considering LDA score ≥ 2 (Segata et al., 2011). In LEfSe analysis, drought treatment (Drought vs. Control) was defined as the Class, while primer region and study ID were incorporated as Subclasses. This enables to identify taxa that are consistently associated with the drought conditions across different studies while reducing the influence of subclass-specific variation.

### 2.2 Comprehensive survey of drought-responsive bacterial taxa across published literatures

Following the meta-analysis of rice rhizosphere 16S rRNA amplicon datasets, a comprehensive literature survey was conducted to identify bacterial genera consistently reported to confer drought tolerance across diverse crop species. Literature search was performed using Web of Science and Google Scholar with the keywords ‘bacteria’ and ‘drought tolerance’ and ‘plant growth promotion’ to identify relevant studies published up to 2025. A total of 337 studies were retrieved, of which 262 studies fulfilled the predefined inclusion criteria after removing review article (71), proceeding paper (2), book chapter (1) and retracted publication (1). Studies were selected based on the following criteria: (i) the study experimentally demonstrated or reported enrichment of a specific bacterial genus under drought conditions or its direct inoculation effect on host plant drought tolerance, (ii) the reported genus had characterized functional mechanisms such as phytohormone modulation, antioxidant enzyme enhancement, osmolyte accumulation, or upregulation of drought-responsive genes; and (iii) the findings were supported by *in-vitro*, greenhouse, or field-based experimental evidence. Studies only reporting changes in microbial community composition without evaluating their underlying functional mechanisms were not considered.

The literature survey identified several bacterial genera reported to enhance drought tolerance in crop plants. For the present study, only those genera represented by well-characterized rice-rhizosphere isolates exhibiting key plant-growth promoting traits and maintained in our laboratory culture collection were selected. Accordingly, representative isolates belonging to the genera *Streptomyces*, *Burkholderia*, *Pantoea*, *Pseudomonas*, and *Variovorax* were used for pot-experimental validation. The selected genera, along with their reported drought-associated functions and studied host-plants, are summarized in **Table 1**. Further details for **Table 1** are provided in **Supplementary Table 2**.

**Table 1:** Drought-associated bacterial genera selected for the present study and their reported functional roles in drought tolerance.

Bacterial genera	Reported drought tolerance mechanisms	References
*Streptomyces*	Enrichment under drought conditions; enhancement of antioxidant defense, root development, phytohormone signaling, and drought-responsive gene expression.	Xu et al., 2018; Li et al., 2020; Pang et al., 2024; Villafane et al., 2024; Li, J. et al., 2025
*Burkholderia*	Improves water-use efficiency, chlorophyll retention, nutrient uptake, ROS detoxification, and regulation of stress-responsive genes and phytohormones.	Naveed et al., 2014; Sheibani-Tezerji et al., 2015; Tallapragada et al., 2016; Huang et al., 2017
*Pantoea*	Enhances osmotic adjustment, antioxidant defense, root growth, exopolysaccharide production, VOC-mediated signaling, and activation of stress-related metabolic pathways.	Chen et al., 2017; Cherif-Silini et al., 2019; Sun et al., 2020; Tariq et al., 2025
*Pseudomonas*	Promotes osmolyte accumulation, antioxidant enzyme activities, water-use efficiency, and expression of drought-responsive transcription factors and stress-related genes.	Sandhya et al., 2010; Sarma and Saikia, 2014; Tiwari et al., 2016; Yasmin et al., 2022
*Variovorax*	Modulates ethylene signaling, phytohormone balance, aquaporin expression, antioxidant defense, and osmolyte accumulation; associated with drought-adapted rhizosphere communities.	Chen et al., 2013; Chandra et al., 2019; Kim et al., 2020; Qi et al., 2022

### 2.3 *In-vitro* screening of selected bacterial strains for plant growth promoting traits

Representative strains of bacterial genera having drought tolerance and plant-growth promoting traits, identified through meta-analysis and documented in the existing literature (**Table 1**; **Supplementary Table 2**), were selected from previously isolated rice-rhizosphere bacterial culture stocks maintained in our laboratory. Taxonomic identities of selected isolates were reconfirmed by phylogenetic analysis, and further tested for different plant-growth promoting traits in triplicate.

#### 2.3.1 Indole-3 acetic acid production

IAA production was assessed following the method outlined by Gordon and Weber (1951). Selected isolates were cultured in nutrient broth supplemented with L-tryptophan (1 mg ml^-^¹) under continuous shaking condition for 72 hrs at 30°C. Supernatant collected were then added with Salkowski’s reagent (2:1, v/v), followed by a 25 mins incubation in dark at room temperature. Development of pink to red color indicates IAA production. Optical density was measured at 530 nm using a UV-Vis spectrophotometer. IAA concentration was estimated using a standard curve generated with known IAA concentrations (μg ml^-^¹).

#### 2.3.2 ACC-deaminase production

ACC deaminase activity of selected isolates was evaluated following the method described by Penrose and Glick (2003). For qualitative estimation, bacterial isolates were screened on Dworkin and Foster’s (Dworkin and Foster, 1958) media amended with (i) 3 mM ACC as sole nitrogen source, (ii) NH_4_Cl as positive control and (iii) nitrogen free media as negative control plates. Growth of isolates on ACC-amended plates after incubation at 28 ± 2 °C for 72 hrs was considered positive for ACC-deaminase activity. For quantitative estimation of α- ketobutyrate production, bacterial isolates were cultured on broth and the supernatant were collected and the absorbance was measured at 540 nm using a UV-Vis spectrophotometer. The ACC deaminase production was calculated using a standard alpha-ketobutyrate calibration curve of known concentration.

#### 2.3.3 Phosphate solubilization activity

Phosphate solubilization activity was assessed qualitatively through spot inoculation of selected isolates on Pikovskaya’s agar plates. Isolates showing clear halo zone formation depicts positive activity (Kucey, 1983).

### 2.4 *In-vitro* drought tolerance screening and compatibility test among selected bacterial isolates

Drought tolerance of selected isolates was evaluated using a polyethylene glycol (PEG) induced osmotic stress assay. Briefly, freshly grown bacterial cultures were inoculated into tryptic soy broth (TSB) supplemented with 25% (w/v) PEG 6000 (- 1.25 MPa), while TSB without PEG 6000 is considered as control. The drought-tolerance categories (OD_600_< 0.30, completely sensitive; 0.30-0.39, sensitive; 0.40-0.50, tolerant; >0.50, completely tolerant) were adopted from Nishu et al. (2022), that measured the thresholds after 24 hrs of incubation. This criterion of growth monitoring was used as a preliminary screening framework for comparing bacterial performance under PEG-induced osmotic stress. As the selected isolates exhibited different intrinsic growth kinetics, the complete growth profile was calculated after (96 hrs) under PEG and control conditions. Subsequently drought response of each isolate was evaluated. The assay was performed using three independent biological replicates.

Furthermore, compatibility tests among selected isolates were conducted according to the protocol outlined by Mikiciński et al. (2016). Briefly, the bacterial strains were co-cultured on nutrient agar plates and zones of inhibition were assessed to observe antagonistic effects. The experiment was repeated thrice to ensure compatibility among selected isolates and optimize consortium formulation prior to soil application.

### 2.5 Experimental site and growth conditions

The pot experiments reported in this study was conducted during the month of May-October of 2025 in a naturally ventilated greenhouse at the Department of Botany, Gauhati University, Assam-India (26°09′ N, 91°40′ E). Preliminary trials were conducted during May-October growing seasons of 2023 and 2024 for optimizing the experimental procedures and verify the reproducibility of the morphological responses under drought. The study was designed to evaluate the potential of rice rhizosphere-associated bacterial isolates on drought tolerant (CR Dhan 802) and drought susceptible (Ranjit) rice varieties under water deficit condition. Plants were grown in plastic pots (17 cm diameter × 15 cm height) containing rice field soil: vermicompost (3:1) mixture under a natural photoperiod with temperature 30 ± 4°C and relative humidity of 80-90%. The soil mixture was thoroughly homogenized to ensure uniform initial soil conditions across all experimental treatment groups. The soil used for the study was collected from agricultural field in Borjhar located near Garal, Kamrup Metropolitian district, Assam-India. The soil exhibits clay loam textures with a slightly acidic pH of 5.75 and an electrical conductivity (EC) of 235 μs/cm. The soil contained organic C of 2.87%, available nitrogen of 222 kg/ha, available phosphorus of 29.23 kg/ha, and available potassium of 198.3 kg/ha.

Rice seeds of two varieties were collected from ICAR-NRRI, Regional Rainfed Lowland Rice Research Station (RRLRRS), Gerua, Assam. Seeds were surface sterilized with 1% sodium hypochlorite (NaClO) for 3 min, rinsed thrice with sterile distilled water (Shukla et al., 2012), and sown in plastic trays (60 × 30 cm) containing rice field soil for germination.

### 2.6 Assessing the effect of selected bacteria on contrasting rice varieties under drought stress

Two contrasting rice varieties CR Dhan 802 (drought-tolerant) and Ranjit (drought-susceptible) were used for the experimental analysis using selected bacterial isolates. The experimental treatments include individual application of all five selected strains, *Streptomyces jiujiangensis* NA-06, *Burkholderia cepacia* NA-07, *Pantoea dispersa* NA-08, *Pseudomonas* sp. NA-15, and *Variovorax guangxiensis* NA-16, with three groups of consortia and a control (Ck) group, which were independently evaluated for each rice variety. Compatible isolates were grouped into two different consortia, Consortium-I (*S*. *jiujiangensis* NA-06, *B. cepacia* NA-07 and *P. dispersa* NA-08), and Consortium-II (*Pseudomonas* sp. NA-15 and *V. guangxiensis* NA-16), and all the selected isolates were combined to form Consortium-III. Bacterial isolates were cultured in nutrient broth and harvested by centrifugation at 5000 rpm for 10 min at 15 °C. The bacterial cell pellets were washed twice with sterile distilled water for the removal of residual culture medium. Washed cell pellets were re-suspended in sterile distilled water. For each treatment, a pot containing approximately 3 kg of soil mixture received 20 ml of bacterial cell suspension adjusted to a cell density of 10^8^ CFU ml^-^¹. For preparation of consortia, bacterial suspensions of individual isolates at a cell density of 10^8^ CFU ml^-^¹ were mixed in equal proportions (Mahreen et al., 2023). An equal volume of sterile water was applied to the control pots.

Following bacterial inoculation, twenty-one-day-old germinated seedlings of uniform height were transferred to their respective pots and maintained up to 45 days under well-watered condition. For each treatment, six biological replicates were considered, of which three were maintained under well-watered conditions and the remaining three were subjected to drought stress. Drought stress was imposed by withholding irrigation under controlled conditions. Stress symptoms were monitored using the International Rice Research Institute (IRRI) Standard Evaluation System (IRRI, 1996). Drought stress induced changes in plant-physiological and biochemical parameters were evaluated when soil moisture content of the respective control pots reached ~20% gravimetric soil moisture content to ensure uniform stress intensity in both the varieties.

### 2.7 Assessment of plant physiological and biochemical parameters

To study the bacterial inoculation effect on selected rice varieties under both drought stressed and non-stressed conditions, plant morpho-physiological and biochemical parameters were evaluated at the sampling stage. Shoot length (SL) was measured in centimeters (cm) from the tip of the longest leaf to the base of the stem. Plants were carefully harvested, separated into shoots and roots, and oven-dried at 70 °C for 48 hrs to determine the dry biomass (Zia et al., 2021). Physiological and biochemical parameters for three biological replicates per group were evaluated following standard protocols.

#### 2.7.1 Determination of relative water content

One representative plant was selected from each pot (biological replicate) for the determination of leaf relative water content (RWC). A 7 cm-long leaf segment was collected from the apical region of a fully expanded leaf from the main tiller of the plant for RWC measurement. Immediately, the fresh weight of the leaf segments was measured, and samples were placed in 15 ml falcon tubes containing distilled water for overnight to determine the leaf turgid weight. Thereafter, the samples were oven-dried at 70 °C for 72 hrs to determine the leaf dry weight (Hoang et al., 2019), using the formula given by Smart and Bingham (1974):


$$\text{RWC}\%=\frac{\left(\text{Fresh Weight}-\text{Dry Weight}\right)}{\left(\text{Turgid Weight}-\text{Dry Weight}\right)}\times 100$$

#### 2.7.2 Determination of total chlorophyll and carotenoid content

The quantitative estimation of total chlorophyll and carotenoid content was determined following the protocol outlined by Arnon (1949). Briefly, 1 g of leaf sample was homogenized with 10 ml of 80% acetone, and centrifuged for 5 mins at 5000 rpm. The supernatant was adjusted to 100 ml in a volumetric flask, and the absorbance of the samples were measured at 480, 510, 645, and 663 nm. Total chlorophyll and carotenoid content were calculated using the following formulae:


$$\text{Total chlorophyll content}\left(\text{mg/g FW}\right)=\frac{\left(20.2\times {\text{A}}_{645}+8.02\times {\text{A}}_{663}\right)\times \text{V}}{1000\times \text{W}}$$

$$\text{Carotenoid content}\left(\text{mg/g FW}\right)=\frac{\left(7.6{\text{A}}_{480}-1.49{\text{A}}_{510}\right)\times \text{V}}{1000\times \text{W}}$$

where A = absorbance at a specific wavelength

V = final volume of extract in 80% acetone

W = fresh weight (FW) of the tissue extracted

#### 2.7.3 Determination of enzymatic and non-enzymatic anti-oxidative stress markers

For the quantitative determination of enzymatic antioxidants, enzyme extracts were prepared following standard protocol. Superoxide dismutase (SOD) activity was determined according to Dhindsa et al. (1981) by measuring the inhibition of photochemical reduction of nitro-blue tetrazolium (NBT) at 560 nm. For catalase (CAT) activity, the decrease in absorbance at 240 nm was measured, which directly corresponds to the decomposition of hydrogen peroxide (H_2_O_2_) (Aebi, 1984). The enzymatic antioxidants were expressed as U min^-1^ g^-1^ FW. Non-enzymatic antioxidants, such as total phenol content (TPC) was spectrophotometrically quantified using Folin-Ciocalteu (FC) method described by Ainsworth and Gillespie (2007) and expressed as mg GAE g^-1^ FW, while proline content was determined using the acid-ninhydrin assay following Bates et al. (1973) and expressed as µmol g^-1^ FW.

#### 2.7.4 Determination of oxidative stress markers

Oxidative stress markers were assessed by quantifying the ROS-H_2_O_2_ accumulation using Vijayaraghavareddy et al. (2017) and lipid peroxidation was estimated in terms of malondialdehyde (MDA) using thiobarbituric acid (TBA) reaction as described by Heath and Packer (1968). The amount of oxidative stress markers accumulation was expressed as µmol g^-1^ FW.

### 2.8 Assessment of soil health parameters

Rhizosphere soil samples were collected in sterile falcon tubes from each biological replicate and stored at -20°C until enzymatic assays were performed. Soil acid phosphatase (ACP) activity was assessed using p-nitrophenyl phosphate (pNPP) as a substrate following Eivazi and Tabatabai (1977). The release of p-nitrophenol was spectrophotometrically quantified at 400 nm, and enzyme activity was expressed as μg p-nitrophenol g^-^¹ dwt h^-^¹. Dehydrogenase (DHA) activity was assessed following Thalmann (1968), using 2,3,5-triphenyltetrazolium chloride (TTC) as an electron acceptor and amount of triphenyl formazan (TPF) was spectrophotometrically quantified at 485 nm. Enzymatic activity of dehydrogenase was expressed as TPF μg^-1^ dwt^-1^ 24h^-1^. Urease (URE) activity was assessed using urea as a substrate, and the amount of released ammonia was measured at 690 nm following Kandeler and Gerber (1988), and urease enzyme activity was expressed as μg NH_4_^+^ N g^-^¹ dwt 2h^-1^.

### 2.9 Statistical analysis

All statistical analyses were performed using the R environment (v4.5.2) (R Core Team, 2025). For each rice variety, physiological, biochemical and soil enzymatic activities data were analyzed separately using two-way ANOVA, considering conditions and bacterial treatment as fixed factors. Differences among treatment means were compared using Tukey’s HSD test at *p* < 0.05. Principal component analysis (PCA) was performed as an exploratory visualization on Z-score-transformed treatment means, to evaluate the multivariate relationship between plant and soil functional traits under drought stress. For the visualization of the relationships among measured parameters and treatment groups, Pearson correlation analysis was done using ‘pheatmap’ (Kolde, 2019) package, while bar graphs and relative abundance boxplots were generated using the ‘ggplot2’ (Wickham, 2016) package in R. For microbiome data, differential abundance analysis was performed at genus level using linear discriminant analysis Effect Size (LEfSe) (Segata et al., 2011) considering LDA score ≥ 2. Features having relative abundance higher than 0.01% were retained and visualized through box plot.

## 3 Results

### 3.1 Drought stress induced shift in bacterial community structure

**Figure 1:**
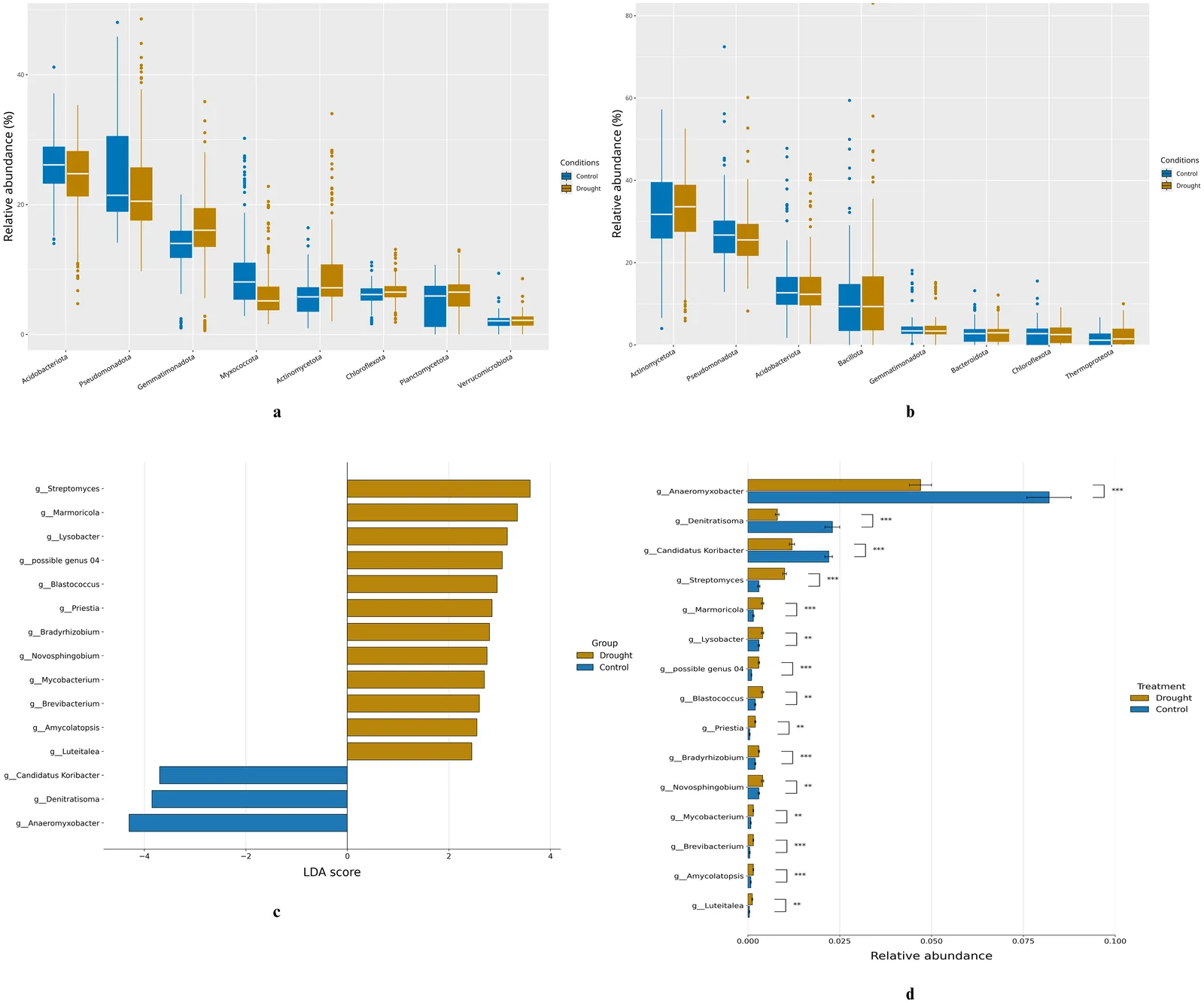
Comparative analysis of rice rhizosphere bacterial communities under drought conditions using global meta-analysis of publicly available 16S amplicon datasets. The sequence files were downloaded from various data repositories like NCBI, and GSA. The boxplot showing the relative abundance (%) of the dominant bacterial phyla in the **(a)** rhizosphere and **(b)** bulk soil under control and drought conditions. In each boxplot, the central line and box edge lines represents the median and interquartile range (IQR; 25^th^-75^th^ percentile) respectively. The whiskers extend to the most extreme observations within 1.5 × IQR, and points beyond the whiskers are displayed as outliers. The differential abundance analysis profile of bacterial communities at genus level identified using linear discriminant analysis effect size (LEfSe) considering LDA score ≥ 2 in the **(c)** rhizosphere under control and drought conditions. However, no bacterial taxa were significantly enriched under drought in bulk soil samples. The relative abundances of differentially abundant bacterial genera were also calculated and represented in horizontal bar plots for **(d)** rhizosphere.

To elucidate the drought stress response pattern of rice rhizosphere bacterial community, we compared the differences in rice rhizosphere communities across a total of 14 independent publicly available rice microbiome datasets. Among these, five studies included both rhizosphere and bulk soil samples, while five included only rhizosphere samples and four included only bulk soil samples (**Supplementary Table 1**). The bacterial compositional analysis of rice rhizosphere under drought stress revealed differences in taxonomic pattern compared to control condition. The phyla Acidobacteriota and Pseudomonadota were most dominant under both control and drought stress conditions. However, under drought phylum Acidobacteriota exhibited a slight reduction in relative abundance compared to the control condition; while the phylum Pseudomonadota maintained comparable abundance under both the conditions, showing greater dispersion under drought. Beyond the aforementioned phyla, Gemmatimonadota, and Actinomycetota exhibited higher relative abundance in the rhizosphere under drought (**Figure 1a**). Differential abundance analysis further revealed that 12 genera were significantly enriched in the rhizosphere under drought-stressed conditions. Out of these 12 genera, six were primarily affiliated with the phylum Actinomycetota, three with the phylum Pseudomonadota, and one each with Bacillota, Acidobacteriota and Fibrobacterota. Among these highly abundant taxa *Streptomyces*, *Marmoricola*, and *Lysobacter* were most prevalent in rice rhizosphere under drought (**Figure 1c**; **Supplementary Table 3**). Furthermore, relative abundance analysis of the differentially abundant bacterial genera in the rhizosphere showed significantly higher abundance of *Streptomyces, Marmoricola, Lysobacter*, *Blastococcus, Priestia, Bradyrhizobium, Novosphingobium*, *Mycobacterium, Brevibacterium, Amycolatopsis* and *Luteitalea* under drought. On the other hand, genera like *Anaeromyxobacter, Denitratisoma*, and *Candidatus Koribacter* showed higher relative abundance under control condition (**Figure 1d**). The taxa identified through this meta-analysis represent candidate rice rhizosphere-associated drought enriched genera, although their associations require further validation under standardized experimental conditions.

Moreover, compositional analysis of bulk soil associated bacterial taxa showed high relative abundance of phyla Actinomycetota, Bacillota, Gemmatimonadota, Bacteroidota, Chloroflexota, and Thermoproteota under drought whereas, Pseudomonadota showed comparatively higher abundance in control condition (**Figure 1b**). Notably, differential abundance analysis further demonstrates that no bacterial taxa were significantly enriched under drought in bulk soil samples.

### 3.2 Characterization of selected drought-associated rice-rhizobacteria

A total of five bacterial strains belonging to the genera *Streptomyces*, *Burkholderia*, *Pantoea*, *Pseudomonas* and *Variovorax* were used in this study (**Table 2**), and the phylogenetic tree showing evolutionary relationships among the isolates is illustrated in **Supplementary Figure 1**. Meta-analysis of rice rhizosphere associated 16S rRNA amplicon datasets further reveals that the genera *Streptomyces* (Actinomycetota) showed significant enrichment under drought. Other representative bacterial isolates were selected based on their well-documented plant growth promotion (PGP) and drought tolerance traits in existing literature (**Table 1**).

**Table 2:** Identities of selected rice rhizosphere-associated bacterial isolates.

Isolate codes	16S rRNA sequence similarity with	Phyla	GenBank accession no.	% Similarity
NA-06	*Streptomyces jiujiangensis*	Actinomycetota	PX149150	99.29
NA-07	*Burkholderia cepacia*	Pseudomonadota	PX149151	99.82
NA-08	*Pantoea dispersa*	Pseudomonadota	PX149152	99.79
NA-15	*Pseudomonas* sp.	Pseudomonadota	PX149153	99.66
NA-16	*Variovorax guangxiensis*	Pseudomonadota	PX149154	99.89

Functional characterization under *in-vitro* conditions revealed variable plant-growth promoting activities among selected bacterial isolates (**Supplementary Table 4**). Higher indole-3-acetic acid (IAA) (34.50 ± 3.75 µg ml^-1^) was produced by *V. guangxiensis* NA-16, followed by *S. jiujiangensis* NA-06 (32.58 ± 1.71 µg ml^-1^) and *P. dispersa* NA-08 (31.20 ± 0.97 µg ml^-1^). Notably, *P. dispersa* NA-08 exhibited the highest 1-aminocyclopropane-1-carboxylate (ACC) deaminase production (35.08 ± 1.72 µmol α-ketobutyric acid mg^-^¹ h^-^¹). Additionally, the highest P solubilization index was shown by *B. cepacia* NA-07 (3.34 ± 0.17), followed by *P. dispersa* NA-08 (3.31 ± 0.13).

Furthermore, *in-vitro* screening of drought tolerance using 25% PEG after 24hrs of incubation revealed that, the selected bacterial isolates exhibited different growth responses. Among the tested isolates, *P. dispersa* NA-08 exhibited the highest growth under PEG-induced *in vitro* drought stress, with an OD_600_ of 0.452 ± 0.038 after 24 hrs, followed by *B. cepacia* NA-07 (0.323 ± 0.007). In contrast, *Pseudomonas* sp. NA-15 and *V. guangxiensis* NA-16 showed comparatively lower growth, with OD_600_ values of 0.173 ± 0.006 and 0.247 ± 0.005, respectively, after 24 hrs under PEG-induced drought stress. After 96 h of incubation, *B. cepacia* NA-07 showed highest growth under PEG treatment (OD_600_ = 0.839 ± 0.026), whereas *S. jiujiangensis* NA-06, a characteristically slow-growing bacterium, exhibited a gradual increase in growth throughout the incubation period, reaching an OD_600_ of 0.140 ± 0.029 under PEG treatment at 96 hrs, compared with 0.357 ± 0.010 in the corresponding control (**Supplementary Table 5**).

### 3.3 Effect of bacterial inoculation in contrasting rice varieties under drought stressed condition

**Figure 2:**
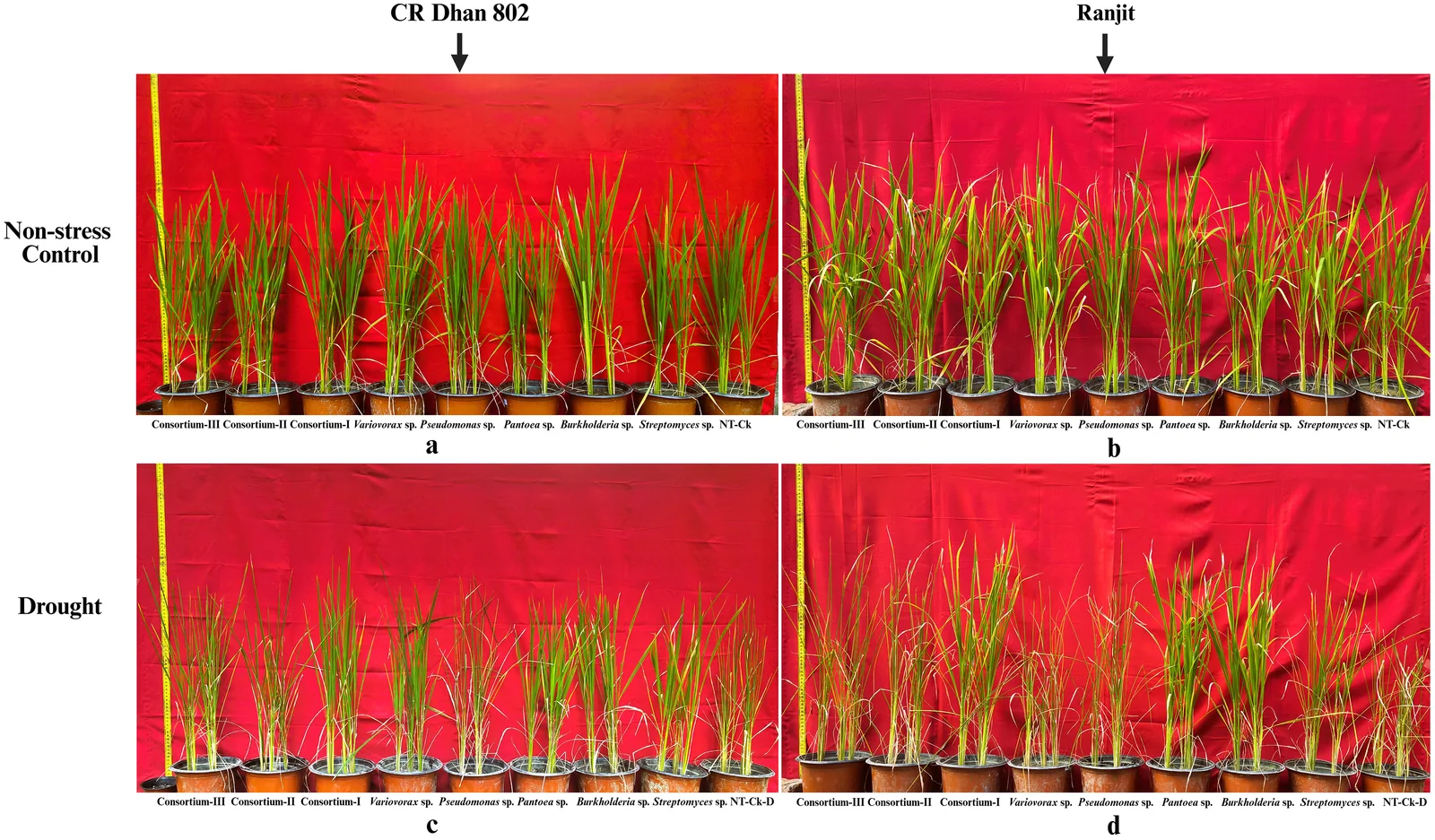
Phenotypic comparison of seedlings treated with selected bacterial isolates individually and in three groups of consortia under **(a, b)** non-stress and **(c, d)** drought stress condition in CR Dhan 802 and Ranjit respectively. Here, Ck: control under non-stress condition, Ck-D: control under drought stress condition, Consortium-I: *S*. *jiujiangensis* NA-06, *B. cepacia* NA-07 and *P. dispersa* NA-08; Consortium-II: *Pseudomonas* sp. NA-15 and *V. guangxiensis* NA-16; Consortium-III: combination of all the selected isolates.

The functional efficacy of selected bacterial isolates was tested by inoculating two rice varieties (susceptible and tolerant) with individual strains and with three consortia groups under both well-watered (control) and drought-stressed conditions (soil moisture ~20%). We observed a significant increase in the growth of rice varieties treated with bacterial isolates compared to the control plants under both non-stress and drought conditions. Drought induced wilting and growth suppression were more prominent in Ranjit compared to CR Dhan 802 (**Figures 2a-d**).

#### 3.3.1 Plant growth and water status

Drought stress significantly reduced shoot length (SL) and shoot dry weight (SDW) in both the rice varieties, with the susceptible variety Ranjit showing a markedly greater decline than the tolerant variety CR Dhan 802. Bacterial inoculation effectively reduced drought-induced growth suppression across both varieties, with a comparatively greater improvement observed in the tested drought sensitive variety Ranjit. In CR Dhan 802, the shoot length (SL) of control plants decreased by 4.8% under drought stress compared with the corresponding control plants under non-stress condition. In contrast, the drought-susceptible variety Ranjit exhibited a much greater reduction, with the shoot length of control plants decreasing by 23.9% under drought relative to its corresponding control plants under non-stress condition. Bacterial inoculation significantly improved SL in both rice varieties under drought conditions, with *B. cepacia* NA-07 treated seedlings exhibiting the highest increase in SL, by 20.4% in CR Dhan 802 and 46.2% in Ranjit than the control group (**Supplementary Figures 2a, b**; **Supplementary Tables 6**, **8**). Notably, plant biomass-SDW was more severely affected by drought than the SL. Drought significantly reduced the SDW of control plants by 42.2% in CR Dhan 802 and 53.2% in Ranjit compared to their control plants under non-stress condition. Seedlings inoculated with *P. dispersa* NA-08 recorded the highest SDW in both the varieties under drought, showing 37.3% and 70.9% increase in SDW of CR Dhan 802 and Ranjit respectively, compared to control plants (interactions in both varieties, *p* < 0.001) (**Supplementary Figures 3a, b**; **Supplementary Tables 6-9**).

Drought stress significantly reduced root dry weight (RDW) in both rice varieties; however, the magnitude of root growth suppression was lower than that observed for SDW. Control plants showed a marked reduction of 10.2% and 15.8% in RDW of CR Dhan 802 and Ranjit, respectively, under drought. Bacterial inoculation significantly alleviated root biomass suppression, with greater improvement observed in drought susceptible rice variety Ranjit. *P. dispersa* NA-08 treated seedlings showed the most effective results compared to the other treatment groups under drought, showing 22.3% and 42.4% increase in RDW of CR Dhan 802 and Ranjit, respectively, over control plants (interactions in both varieties, *p* < 0.001) (**Supplementary Figures 3c, d**; **Supplementary Tables 6**, **8**). Likewise, a significant reduction in leaf RWC was observed in both rice varieties under drought stress; however, the magnitude of reduction was variety-specific and markedly alleviated by bacterial inoculation. Control plants under drought experienced a decline of 54.6% and 77.8% leaf RWC in the varieties CR Dhan 802 and Ranjit respectively. The greater reduction in leaf RWC observed in Ranjit than CR Dhan 802 indicates higher water-status sensitivity of sensitive variety under imposed water-deficit conditions. However, bacterial inoculation substantially mitigated this dehydration effect in both the rice varieties under drought condition. In CR Dhan 802, *P dispersa* NA-08 and *B. cepacia* NA-07 treated seedlings showed the most effective results, maintaining the highest RWC compared to the control plants under drought. Similarly, Ranjit seedlings treated with *P. dispersa* NA-08, *B. cepacia* NA-07, and Consortium-I showed higher RWC compared to the control plants under drought (**Supplementary Figures 3e, f**; **Supplementary Tables 6**, **8**). The interactions between conditions and treatments of CR Dhan 802 and Ranjit showed *p <* 0.001 and *p* = 0.164 respectively (**Supplementary Tables 7**, **9**).

#### 3.3.2 Photosynthetic pigments

**Figure 3:**
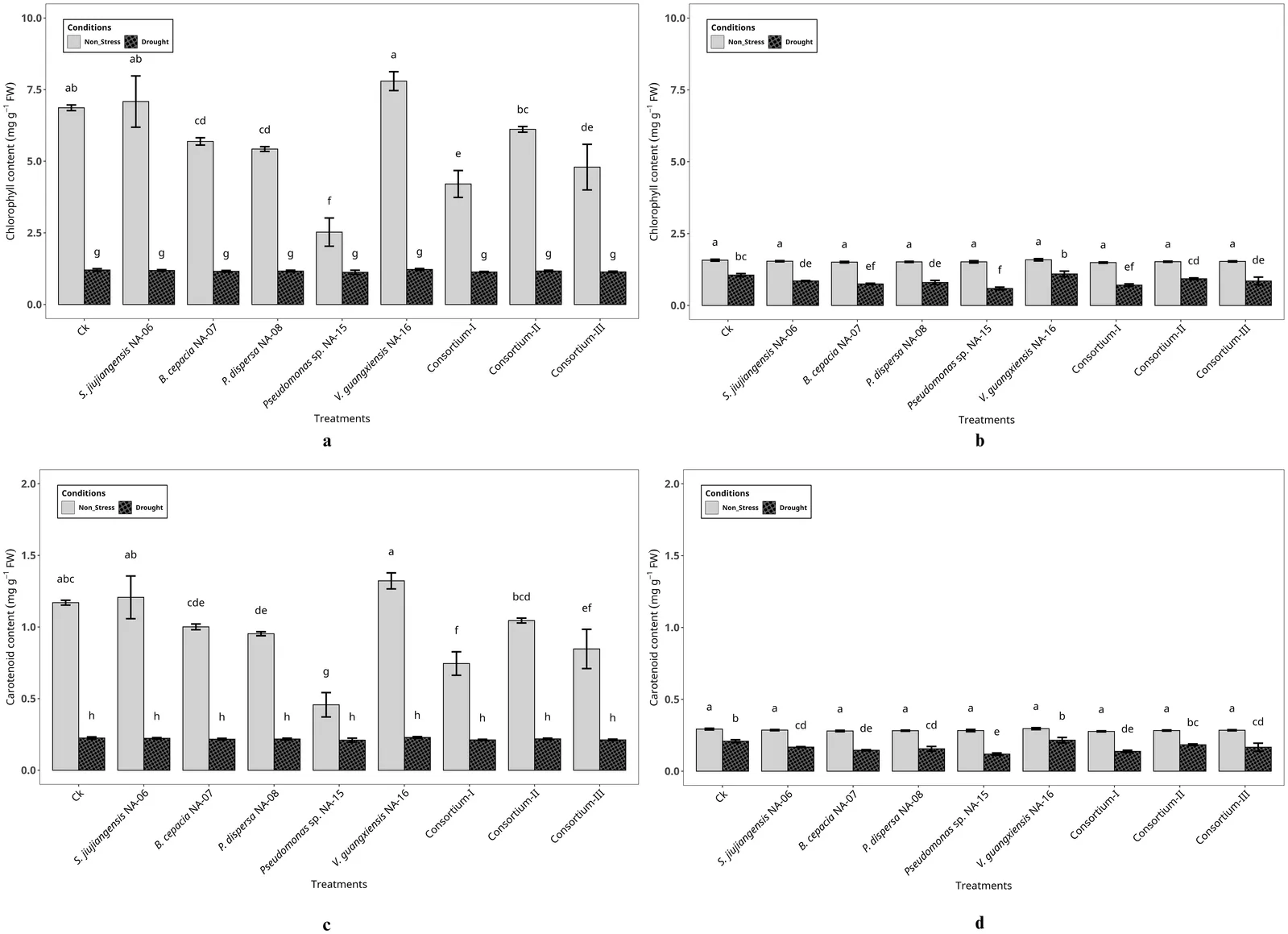
Effect of bacterial treatments (Ck, *S*. *jiujiangensis* NA-06, *B. cepacia* NA-07, *P. dispersa* NA-08, *Pseudomonas* sp. NA-15, *V. guangxiensis* NA-16, Consortium-I, Consortium-II and Consortium-III) on total chlorophyll and carotenoid content of drought susceptible (Ranjit) and tolerant (CR Dhan 802) rice varieties under control and drought conditions. Here, **(a, b)** total chlorophyll, and **(c, d)** carotenoid content of CR Dhan 802 and Ranjit, respectively. Bars represent mean ± standard deviation (SD) of three biological replicates (n = 3). Different letters above bars indicate pairwise significant differences among treatment groups within each condition as determined by Tukey’s HSD *post hoc* test following two-way ANOVA (*p* < 0.05). The interaction effect between conditions and bacterial treatment was significant for total chlorophyll and carotenoid content in both CR Dhan 802 and Ranjit (*p* < 0.001). The corresponding adjusted pairwise comparison p-values are provided in **Supplementary Tables 7**, **9**.

We observed a significant decrease in the total chlorophyll content of both varieties under drought condition. In CR Dhan 802, there was no significant difference observed in total chlorophyll content among the treated seedlings and the control under drought stress condition. However, bacterial inoculation significantly enhanced the chlorophyll content in non-stress condition (interactions *p* < 0.001; **Figure 3a**; **Supplementary Table 6**). Contrastingly, in Ranjit the seedling treated with *V. guangxiensis* NA-16 (1.1 ± 0.1 mg g^-1^FW) showed improved in total chlorophyll content compared to the control under drought (*p* < 0.001 for all factors; **Figure 3b**; **Supplementary Table 8**). In addition, the same trend was observed in carotenoid content, where no significant difference was found among bacteria treated seedlings and control in CR Dhan 802 under drought. However, bacterial inoculation significantly enhanced the carotenoid content in non-stress condition (*p* < 0.001; **Figure 3c**; **Supplementary Table 6**). In contrast, bacteria treated seedlings showed no significant difference in carotenoid content among treated seedlings and control group under non-stress condition in Ranjit (*p* < 0.001 for all factors; **Figure 3d**; **Supplementary Table 8**).

#### 3.3.3 Enzymatic and non-enzymatic antioxidant responses

**Figure 4:**
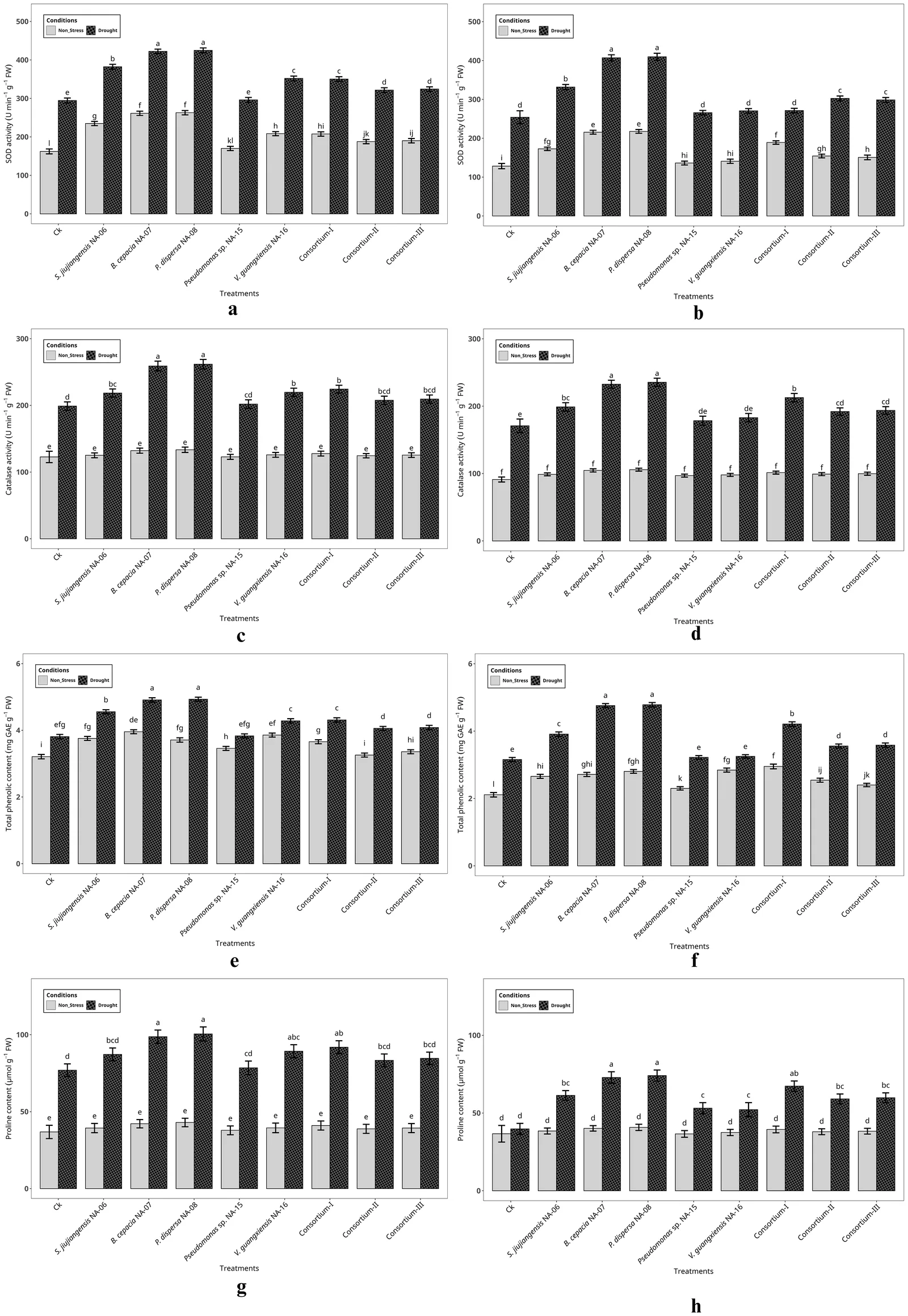
Effect of bacterial treatments (Ck, *S*. *jiujiangensis* NA-06, *B. cepacia* NA-07, *P. dispersa* NA-08, *Pseudomonas* sp. NA-15, *V. guangxiensis* NA-16, Consortium-I, Consortium-II and Consortium-III) on superoxide dismutase (SOD), catalase (CAT), total phenol content (TPC) and proline content of drought susceptible (Ranjit) and tolerant (CR Dhan 802) rice varieties under control and drought conditions. Here, **(a, b)** SOD and **(c, d)** CAT **(e, f)** TPC and **(g, h)** proline content of CR Dhan 802 and Ranjit, respectively. Bars represent mean ± standard deviation (SD) of three biological replicates (n = 3). Different letters above bars indicate pairwise significant differences among treatment groups within each condition as determined by Tukey’s HSD *post hoc* test following two-way ANOVA (*p* < 0.05). The interaction effect between conditions and bacterial treatment was significant for SOD, CAT, TPC and proline in both CR Dhan 802 and Ranjit (*p* < 0.01). The corresponding adjusted pairwise comparison p-values are provided in **Supplementary Tables 7**, **9**.

Drought significantly upregulated enzymatic antioxidants such as superoxide dismutase (SOD) and catalase (CAT) activities in both varieties. Bacterial inoculation further modulated enzymatic antioxidant responses in rice plants, suggesting enhanced regulation of oxidative stress under drought conditions. Both SOD and CAT activities were found to be elevated in treated seedlings compared to the control plants in both the varieties under drought, with *P. dispersa* NA-08 and *B. cepacia* NA-07 showing the highest enzymatic activities. In CR Dhan 802, the elevation of SOD activity under these two treatments was 44% more than that of the control plants under drought. A similar trend was also observed in CAT activity. Moreover, similar responses were also observed in the case of Ranjit, however the absolute SOD and CAT values were lower than that of CR Dhan 802 under drought conditions. Two-way ANOVA indicated significant effects of conditions, treatments, and their interaction on both SOD and CAT activities (*p* < 0.001 for all factors) in both the varieties (**Figures 4a-d**; **Supplementary Tables 6-9**).

The total phenol content (TPC) and proline content increased significantly in both varieties of bacteria-inoculated seedlings compared to the control plants. The magnitude of the increase in TPC and proline content in inoculated seedlings was variety-specific, with Ranjit showing a proportionally greater drought response than the tolerant variety CR Dhan 802. Seedlings treated with *P. dispersa* NA-08 followed by *B. cepacia* NA-07 showed highest TPC (4.93 ± 0.06 mg GAE g-1 FW and 4.91 ± 0.07 mg GAE g-1 FW, respectively) and proline (100.50 ± 4.56 µmol g^-1^ FW and 98.70 ± 4.32 µmol g^-1^ FW, respectively) accumulation in CR Dhan 802 compared to the control plants under drought. Similar responses were also observed in Ranjit with Consortium-I showing notable improvements in TPC and proline content compared to the control plants under drought (*p<* 0.01) (**Figures 4e-h**; **Supplementary Tables 6-9**).

#### 3.3.4 Responses of oxidative stress markers

**Figure 5:**
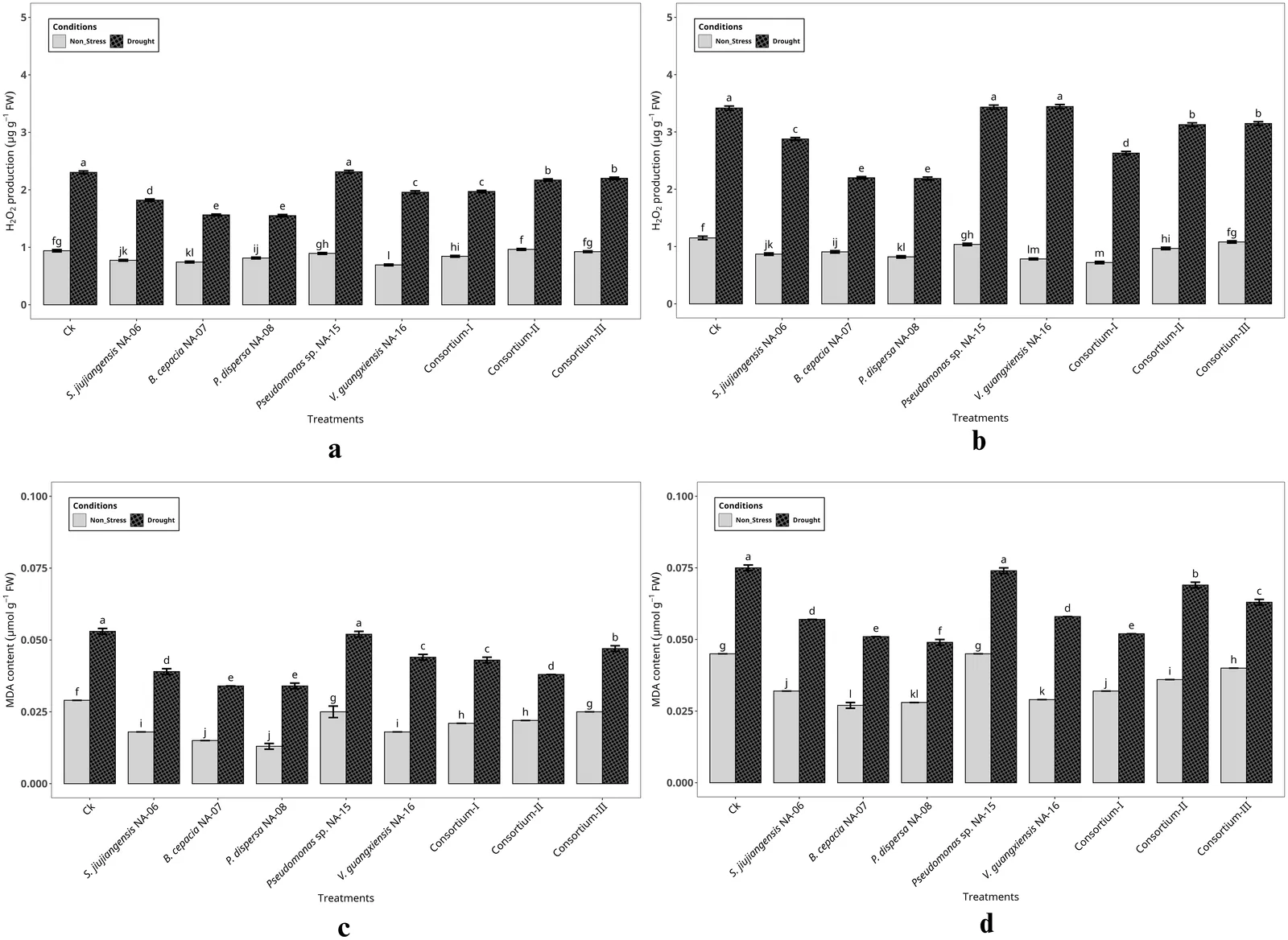
Effect of bacterial treatments (Ck, *S*. *jiujiangensis* NA-06, *B. cepacia* NA-07, *P. dispersa* NA-08, *Pseudomonas* sp. NA-15, *V. guangxiensis* NA-16, Consortium-I, Consortium-II and Consortium-III) on hydrogen peroxide (H_2_O_2_) and malondialdehyde (MDA) content of drought susceptible (Ranjit) and tolerant (CR Dhan 802) rice varieties under control and drought conditions. Here, **(a, b)** H_2_O_2_ **(c, d)** MDA of CR Dhan 802 and Ranjit respectively. Bars represent mean ± standard deviation (SD) of three biological replicates (n = 3). Different letters above bars indicate pairwise significant differences among treatment groups within each condition as determined by Tukey’s HSD *post hoc* test following two-way ANOVA (*p* < 0.05). The interaction effect between conditions and bacterial treatment was significant for H_2_O_2_ and MDA in both CR Dhan 802 and Ranjit (*p* < 0.001). The corresponding adjusted pairwise comparison p-values are provided in **Supplementary Tables 7**, **9**.

Drought stress markedly increased both hydrogen peroxide (H_2_O_2_) accumulation and lipid peroxidation in control plants, indicating greater oxidative damage than in most bacterial- treated seedlings in both varieties. However, bacterial inoculation reduced this oxidative damage in rice varieties, although the magnitude of the response varied notably among treated groups. In CR Dhan 802 and Ranjit, seedlings treated with *P. dispersa* NA-08 and *B. cepacia* NA-07 showed the lowest oxidative damage, depicting lower levels of H_2_O_2_ accumulation and malondialdehyde (MDA) content compared to the control plants under drought. In CR Dhan 802, control plants and seedlings treated with *Pseudomonas* sp. NA-15 exhibited significantly higher accumulation of H_2_O_2_ measuring 2.30 ± 0.03 µmol g^-1^FW and 2.31 ± 0.03 µmol g^-1^FW respectively under drought. In contrast, *P. dispersa* NA-08 (1.55 ± 0.02 µmol g^-1^FW) exhibit significantly reduced oxidative damage by accumulating lowest levels of H_2_O_2_ (**Figure 5a**; **Supplementary Table 6**). Moreover, a similar pattern was observed for lipid peroxidation, in which *B. cepacia* NA-07 and *P. dispersa* NA-08 showed the lowest MDA content (0.034 ± 0.001 µmol g^-1^FW), compared to the control group under drought-stressed condition (**Figure 5c**; **Supplementary Table 6**). Seedlings treated with different bacterial consortia showed intermediate reductions, while *Pseudomonas* sp. NA-15 showed no significant difference in MDA content with the control group (*p* < 0.001 for all factors; **Supplementary Tables 7**, **9**). A similar pattern was observed in Ranjit, where drought stress reduced both oxidative stress markers in bacteria-treated seedlings, where the lowest H_2_O_2_ content of 2.19 ± 0.03 µmol g^-1^FW and MDA content of 0.049 ± 0.001 µmol g^-1^FW accumulation was observed in *P. dispersa* NA-08 treated seedlings. In contrast, *Pseudomonas* sp. NA-15 did not differ significantly from the control group for MDA content under drought (*p* < 0.001 for all factors; **Figures 5b, d**; **Supplementary Tables 8**, **9**).

### 3.4 Soil enzyme activities

**Figure 6:**
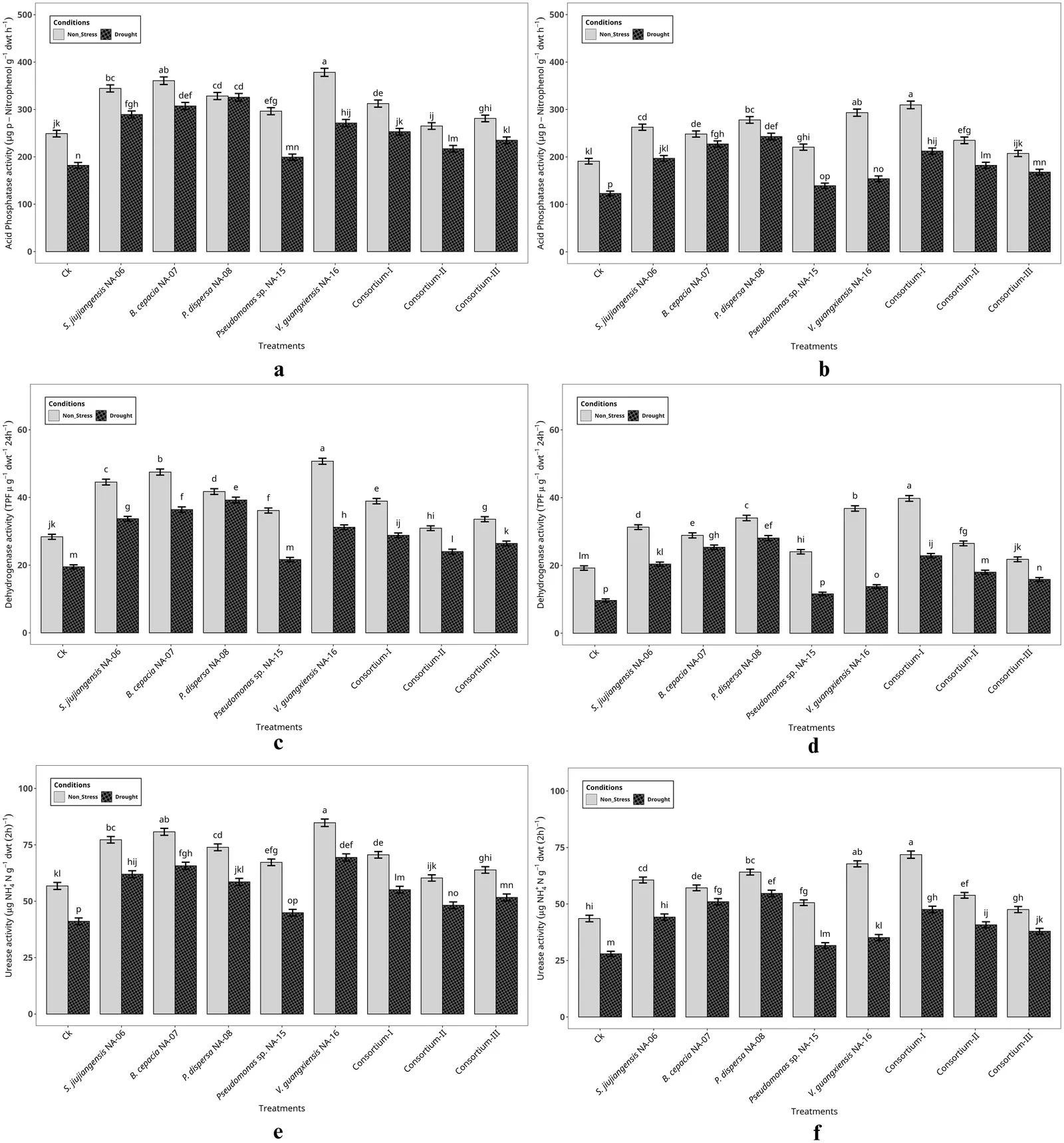
Effect of bacterial treatments (Ck, *S. jiujiangensis* NA-06, *B. cepacia* NA-07, *P. dispersa* NA-08, Pseudomonas sp. NA-15, *V. guangxiensis* NA-16, Consortium-I, Consortium-II and Consortium-III) on soil acid-phosphatase (ACP), dehydrogenase (DHA) and urease (URE) activities of drought susceptible (Ranjit) and tolerant (CR Dhan 802) rice varieties under control and drought conditions. Here, **(a, b)** ACP **(c, d)** DHA and **(e, f)** URE of CR Dhan 802 and Ranjit respectively. Bars represent mean ± standard deviation (SD) of three biological replicates (n = 3). Different letters above bars indicate pairwise significant differences among treatment groups within each condition as determined by Tukey’s HSD *post hoc* test following two-way ANOVA (p < 0.05). The interaction effect between conditions and bacterial treatment was significant for ACP, DHA and URE in both CR Dhan 802 and Ranjit (p < 0.001). The corresponding adjusted pairwise comparison p-values are provided in **Supplementary Table 11**.

A significant reduction in soil acid-phosphatase (ACP), dehydrogenase (DHA) and urease (URE) activities were observed in both the varieties under drought stress condition. The reduction in soil enzymatic activities under drought was comparatively more pronounced in Ranjit than in CR Dhan 802. Under drought, bacterial inoculation was associated with higher soil enzyme activities than the control. In CR Dhan 802, *P. dispersa* NA-08 treated rice seedlings showed the highest ACP activity of 325.67 ± 8.02 µg p-nitrophenol g^-1^dwt h^-1^, whereas control plants recorded the lowest ACP activity of 182 ± 6 µg p-nitrophenol g^-1^dwt h^-1^ under drought. A similar pattern was also observed in Ranjit, but the recorded values were notably lower than those of CR Dhan 802. Under non-stress condition, *V. guangxiensis* NA-16 and *B. cepacia* NA-07 showed the highest ACP activity in CR Dhan 802, while Consortium-I and *V. guangxiensis* NA-16 showed the highest ACP activity in Ranjit (*p* < 0.001) (**Figures 6a, b**; **Supplementary Tables 10**, **11**).

Bacterial inoculation substantially enhanced DHA under drought; where *P. dispersa* NA-08 and *B. cepacia* NA-07 showed the highest activity in both the varieties, while control group recorded the lowest value. Under non-stress condition, *V. guangxiensis* NA-16 exhibited the highest DHA activity in CR Dhan 802, while Consortium-I showed highest DHA activity in Ranjit (*p<* 0.001; **Figures 6c, d**; **Supplementary Tables 9**, **10**). Under drought the highest URE activity of 69.4 ± 1.6 µg NH_4_^+^-N g^-^¹ dwt 2 h^-^¹ was recorded in treatment *V. guangxiensis* NA-16, followed by *B. cepacia* NA-07 (65.7 ± 1.6 µg NH_4_^+^-N g^-^¹ dwt 2 h^-^¹) and lowest was shown in control group (41.1 ± 1.5 µg NH_4_^+^-N g^-^¹ dwt 2 h^-^¹) in the variety CR Dhan 802. However, in Ranjit the highest URE activity was observed in the treatment *P. dispersa* NA-08 (54.67 ± 1.45 µg NH_4_^+^-N g^-^¹ dwt 2 h^-^¹), followed by *B. cepacia* NA-07 and lowest by control group (27.93 ± 1.15 µg NH_4_^+^-N g^-^¹ dwt 2 h^-^¹). Under non-stress condition, highest URE activity was recorded in *V. guangxiensis* NA-16, followed by *B. cepacia* NA-07, and lowest was recorded in control plants in CR Dhan 802. Similarly in Ranjit, the treatment group Consortium-I showed the highest URE activity and control group showed the lowest activity (*p <* 0.001; **Figures 6e, f**; **Supplementary Tables 10**, **11**).

**Figure 7:**
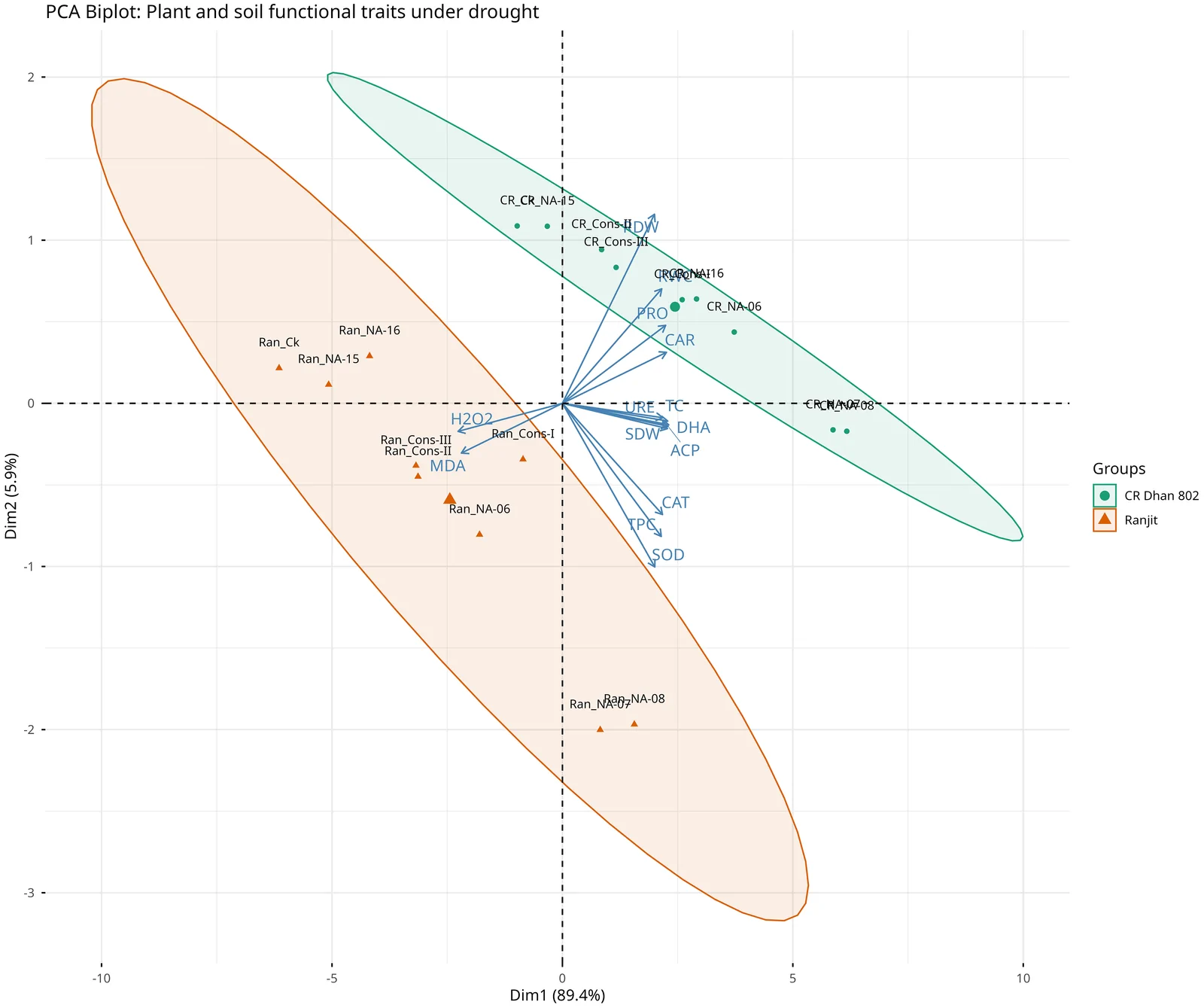
Score plot from the Principal Component Analysis (PCA) of plant physiological and biochemical traits along with soil enzymatic activities of CR Dhan 802 (green dots) and Ranjit (red triangles) under drought stress conditions. Scores represent the mean values of standardized and normalized physiological and biochemical parameters. Here, RDW- root dry weight, SDW- shoot dry weight, CAR- carotenoid content, TC- total chlorophyll content, RWC- relative water content, CAT- catalase activity, H_2_O_2_- hydrogen peroxide content, MDA- malondialdehyde content, TPC- total phenol content, PRO-proline content, SOD- superoxide dismutase, ACP- soil acid phosphatase activity, DHA- soil dehydrogenase activity, URE- soil urease activity. All measured traits were included in the PCA following Z-score transformation to ensure comparability across variables.

As an exploratory multivariate visualization, the PCA biplot shows separation between the two rice varieties, CR Dhan 802 and Ranjit, along the major axes. The data matrix used for the analysis consisted of 18 total observations and 14 variables, where 95.3% of total variance was attributed to the projection (including dimensions 1 and 2) (**Figure 7**). High positive loadings of RDW along Dim 1 were strongly associated with the drought-tolerant variety CR Dhan 802, indicating greater biomass accumulation under water-deficit conditions. This enhanced biomass production was accompanied by higher RWC and osmoprotectant accumulation, exhibiting better drought adaptation. Simultaneously, enzymatic and non-enzymatic variables, notably SOD exhibited strong positive contributions along Dim1, suggesting an enhanced antioxidant defense barrier protecting CR Dhan 802 from oxidative damage. Notably, seedlings treated with *P. dispersa* NA-08 and *B. cepacia* NA-07 in CR Dhan 802 showed strong positive associations with total chlorophyll content, TPC, SOD, CAT, and soil enzyme activities. In contrast, treated seedlings of Ranjit under drought stress exhibited a stronger association with oxidative stress markers, particularly H_2_O_2_ and MDA. However, within the variety Ranjit, seedlings treated with *P. dispersa* NA-08 and *B. cepacia* NA-07, clustered closer to antioxidant-related traits compared to control group, *Pseudomonas* sp. NA-15, and *V. guangxiensis* NA-16 treated samples.

## 4 Discussion

Drought is increasingly threatening global food production due to its rising frequency, severity and temporal extent (Niu et al., 2025). Although the role of plant-associated microbiomes in mitigating drought stress has gained considerable attention, systematic characterization of rice rhizosphere-associated microbes and their functional validation remains limited (Roldan et al., 2025). Despite the well-recognized importance of plant growth promoting rhizobacteria in plant drought tolerance, systematic characterization of rice-rhizosphere associated microbes and their evaluation for rice drought tolerance remains poorly understood. In this context, we evaluate drought tolerance potential of rice-rhizosphere associated bacterial taxa in drought-tolerant and drought-susceptible rice varieties under water deficit condition. We performed a global meta-analysis of bacterial community datasets from rice-rhizosphere and bulk soil samples under drought and control conditions. The results of our meta-analysis revealed that drought responsive bacterial taxa differed markedly between rice rhizosphere and bulk soil samples, highlighting the critical role of the plant in shaping its surrounding microbial niche. However, one limitation of present study is that the meta-analysis integrated various amplicon datasets targeting different hypervariable regions using multiple primer sets. Although, all datasets were processed through a unified bioinformatics workflow to improve comparability, there might have some influences on observed bacterial community composition due primer-specific amplification biases and variation in methodological approaches.

We found that, bacterial phyla such as Gemmatimonadota and Actinomycetota, exhibited the most pronounced enrichment under drought, consistent with previous studies reporting the enrichment of these phyla under drought (Li, X. et al., 2025, Li, J. et al., 2025). Additionally, Acidobacteriota and Pseudomonadota were found as the most abundant phyla under both control and drought conditions. The results of our meta-analysis align with existing literature documenting drought-induced shifts in rhizosphere microbial communities across multiple crop species. These findings are consistent with the broader meta-analysis of drought-associated bacterial communities across 26 crop species conducted by Niu et al. (2025), which similarly reported increased relative abundances of Actinobacteria, Chloroflexi, Firmicutes, and Planctomycetes in the rhizosphere under drought conditions. Furthermore, previous studies from maize rhizosphere support this pattern, showing higher abundance of Acidobacteriota, Actinomycetota, Planctomycetota, and Pseudomonadota under drought stress conditions (Ghosh et al., 2025; Zacamo-Velázquez et al., 2026). Notably, differential abundant analysis further showed twelve drought enriched genera in the rhizosphere soil samples, highlighting the fact that the rhizosphere functions as a highly selective ecological filter. However, no significantly differential bacterial taxa were detected in the bulk soil under drought conditions. Moreover, this interpretation is based on datasets compiled from different studies included in the meta-analysis. This compartment specific enrichment is supported by the observations of a study conducted by Naylor et al. (2017) in 18 different grass species, where they found that bacterial community enrichment is higher within the root tissues than their surroundings under drought. The selective recruitment of drought associated beneficial microbes in the rhizosphere might takes place through changes in root exudation pattern and plant signaling pathways (Fitzpatrick et al., 2020).

We identified significant enrichment of 12 most abundant drought responsive bacterial genera associated with rice rhizosphere namely *Streptomyces, Marmoricola, Blastococcus*, *Mycobacterium* and *Amycolatopsis* under the phylum Actinomycetota; *Lysobacter, Novosphingobium*, and *Bradyrhizobium* under the phylum Pseudomonadota; *Luteitalea* under the phylum Acidobacteriota; *Priestia* under the phylum Bacillota and possible genus 04 under the phylum Fibrobacterota. These bacterial genera were well reported in having different plant-growth promoting traits including ACC-deaminase activity, IAA-production, and phosphate solubilization activity. The relative abundances of Acidobacteriota, Actinomycetota, Planctomycetota, and Pseudomonadota were reported in having different plant-growth promoting traits, suggesting their role in plant growth improvement under water deficient conditions (Guo et al., 2023; Wu et al., 2024; Ghosh et al., 2025). Simultaneously, members of the phylum Actinomycetota, specially the genus *Streptomyces* were reported to be highly abundant in crop rhizosphere under drought (Liu, K. et al., 2025). Similarly, a bacterial genus *Lysobacter* possesses different plant-growth promoting traits (Dai et al., 2023) and was reported to be abundant in rhizosphere of broomcorn millet under drought (Na et al., 2019). Among the drought-enriched genera identified through the meta-analysis, only *Streptomyces* was represented in our rice rhizosphere culture collection and was therefore included in the pot experiments.

We have also performed literature searches across various databases and it revealed that several bacterial genera *Streptomyces, Burkholderia*, *Pantoea*, *Pseudomonas*, and *Variovorax* consistently reported for drought stress mitigation across diverse crop systems (Mahreen et al., 2023; Ferrante et al., 2024; Sharma and Joshi, 2025). Based on these findings, representative rice-rhizosphere associated bacterial isolates from these genera were selected for assessing their drought stress mitigation potential in two rice varieties, namely CR Dhan 802 (drought-tolerant) and Ranjit (drought-susceptible). Plants inoculated with the isolate *P. dispersa* NA-08, maintained higher leaf RWC, photosynthetic pigment stabilization, and accumulate higher plant anti-oxidative enzymes that aids in lowering oxidative stress markers. This finding is supported by previous reports that highlighted the role of genus *Pantoea* in drought stress alleviation in crop plants. For example, Chen et al. (2017), in their study reported that inoculation of *Pantoea alhagi* LTYR-11Z enhances drought tolerance in wheat, via increased accumulation of soluble sugars, lower accumulation of oxidative stress markers such as MDA, and lower leaf chlorophyll degradation. Similar experimental studies conducted by Cherif-Silini et al. (2019) reported that inoculation of durum wheat with *Pantoea agglomerans* improves plant growth under drought and salt stress conditions. The specific role of *P. dispersa* in alleviating drought stress in plants remains poorly characterized. However, the application of plant growth promoting isolate, *P. dispersa* in diverse crop plants showed significant enhancement in plant growth. For example, inoculation of *P. dispersa* showed higher PGP and nitrogenase activity in sugarcane (Singh et al., 2021); enhanced seed germination, seedling vigor index, root growth in rice (Rawat et al., 2022); increase growth and yield in chickpea (Tariq et al., 2023). Furthermore, genome analysis of *P. dispersa* reveals its extensive potential as plant growth promoting rhizobacteria supported by the presence of diverse genetic pathways linked to (PGP), nutrient mobilization and stress adaptation (Hossain, 2024).

Besides *P. dispersa* NA-08, bacterial isolate- *B. cepacia* also exhibited considerable potential in improving drought tolerance in both the rice varieties. Numerous studies have reported the potential of this genus for conferring drought tolerance. For example, inoculation of *Burkholderia phytofirmans* PsJN in maize showed enhancement in leaf area, plant biomass, leaf RWC, chlorophyll content, and photochemical efficiency of PSII resulting higher photosynthesis rate under drought (Naveed et al., 2014). Furthermore, transcriptomic profiling of *B. phytofirmans* PsJN colonizing tomato plants under drought revealed higher expression of genes regulating cellular homeostasis, ROS detoxification, and group IV sigma factors of extra-cytoplasmatic function (ECF). These ECF-sigma factors act as cell surface signaling elements, indicating their role in sensing the changes in surrounding environmental conditions and simultaneously modulating the bacterial metabolic responses (Sheibani-Tezerji et al., 2015). Huang et al. (2017) in another study reported that inoculation of *Burkholderia* sp. ADR10 showed significantly enhanced plant-growth in *Arabidopsis thaliana* and maize under drought. Rhizospheric isolate *B. cepacia* SCAUK0330, possess high phosphate solubilizing activity and showed significant PGP in maize (Zhao et al., 2014). Similarly, de Andrade et al. (2019) in a study documented that, *B. cepacia* (CCMA 0056) exhibiting plant growth promoting traits such as IAA-production, N_2_-fixation, and its co-inoculation with *Enterobacter cloacae* (CCMA 1285) in strawberry, significantly enhance host plant biomass and growth. Additionally, Janaki et al. (2024) reported that a rhizosphere-derived *B. cepacia* strain exhibited multiple plant growth-promoting traits, including phosphate solubilization, IAA, siderophore and hydrogen cyanide (HCN) production, along with cadmium (Cd) and lead (Pb) tolerance abilities. The strain also displayed antifungal activity against *Rhizoctonia solani* and *Fusarium oxysporum*. Inoculation of tomato plants with *B. cepacia* significantly improved seed germination, plant growth, biomass accumulation, and antioxidant defense under Cd and Pb stress, demonstrating its potential to alleviate heavy metal toxicity while promoting plant growth. Although several strains of *B. cepacia* have been reported as beneficial plant growth-promoting bacteria, the *B. cepacia* complex also includes opportunistic human pathogens. Hence, isolate NA-07 should undergo further taxonomic confirmation and biosafety evaluation before field-scale application.

Notably, bacterial inoculation significantly increased plant growth parameters including shoot length, and plant biomass but the magnitude of growth promotion differed between two rice varieties. Under non-stress conditions, we observed that seedlings of both the varieties treated with *V. guangxiensis* NA-16 showed highest SL and RDW. However, highest SDW was observed in seedlings treated with *V. guangxiensis* NA-16 followed by *B. cepacia* NA-07 in CR Dhan 802 whereas, Consortium-I followed by *V. guangxiensis* NA-16 showed highest SDW in Ranjit. The growth promotion effect of *V. guangxiensis* NA-16 has been supported by Finkel et al. (2020), which documented that *Variovorax* helps in the regulation of plant hormone levels, that supports root development, plant growth and modulation of plant associated microbial community. These overall findings demonstrated different microbial effect on contrasting varieties and were further supported by a study conducted by Sharma et al. (2014). In this study, rice genotypes inoculated with different plant growth promoting rhizobacteria showed enhancement in plant growth attributes such as plant biomass, shoot length, and chlorophyll content, but the magnitude of growth promotion differed among rice genotypes.

Beyond plant physiological and biochemical responses microbial inoculation significantly influenced soil enzymatic activities, providing evidence that these microorganisms actively reshape the soil biochemical environment under water deficit conditions. The improvement in soil ACP activity upon bacterial inoculation suggests enhanced phosphorus mobilization through microbes under water deficit condition (Richardson and Simpson, 2011). Similarly, elevation of soil DHA activity reveals presence of active microbial community and their metabolic resilience (Burns et al., 2013). Furthermore, increased soil URE activity represents increased nitrogen mineralization and stable nitrogen transformation (Kalala et al., 2022). These results suggest that bacterial inoculation was associated with short-term enhancement of rhizosphere biochemical activity under drought (Dai et al., 2025). However, long-term effects on soil fertility, nutrient availability, and microbial community composition require further validation. These alterations can play a pivotal role in biogeochemical cycling through phosphorus solubilization and N_2_-fixation thereby enhancing nutrient availability for plants (Li et al., 2024).

## 5 Conclusion

Drought stress restructures the rice rhizosphere microbiome, with enrichment of bacterial genera potentially involved in stress adaptation. A meta-analysis carried out in this study identified highly abundant drought-associated bacterial taxa namely Gemmatimonadota, Actinomycetota, Chloroflexota, Planctomycetota, Verrucomicrobiota and Pseudomonadota in rice rhizosphere. However, differential abundance analysis did not reveal a consistent core genus between rhizosphere and bulk soil, which highlights the dynamic nature of microbial recruitments. Pot experiments using selected bacterial isolates on two contrasting rice varieties showed improved drought-related physiological and biochemical traits, with a comparatively stronger response in the susceptible variety Ranjit. Notably, bacterial inoculation elicited distinct responses under well-watered and drought conditions, with substantially greater improvements observed under drought stress, indicating that the beneficial effects of these isolates are primarily associated with stress adaptation rather than general growth promotion. Overall, the findings provide an insight that, drought associated rhizosphere microbiome plays a significant role in drought stress tolerance. These drought-associated rhizospheric microbial taxa possessing plant-growth promoting and drought tolerance traits represent promising candidates for future development as microbe-based agricultural inputs; however, their field performance, formulation stability, colonization efficiency, biosafety, and impact on crop productivity require further validation. Multi-omics approaches should be utilized for understanding of plant metabolic pathways and stress adaptive traits of drought enriched microbial taxa. This will facilitate the successful bio formulation of synthetic microbial consortia with enhanced functional reliability and translational potential. Additionally, field-level validation of these potent microbial formulations will be essential to evaluate their ecological stability and performance consistency under diverse ecological complexes.

## Data Availability

The original contributions presented in the study are included in the article/**Supplementary Material**. Further inquiries can be directed to the corresponding author.
