# Supplementary material for: Global meta-analysis of drought associated rice rhizosphere microbiome data and soil application of selected bacteria reveals potential of Pantoea dispersa for drought stress alleviation in rice plants
Source: Front Plant Sci. 2026 Sep 3;17:1904089. doi: 10.3389/fpls.2026.1904089 (PMC13581755; doi:10.3389/fpls.2026.1904089)
Supplement: Supplementary file 1 [file SupplementaryFile1.docx]

**Supplementary Information**

**List of Figures:**

1. **Supplementary Figure 1.** Phylogenetic tree illustrating the evolutionary relationships of the selected rice rhizospheric bacterial isolates based on 16S rRNA gene sequences. The tree was constructed using the Maximum likelihood method with 1000 bootstrap replications. Bootstrap values (>30%) are indicated at the branch nodes. The isolates obtained in this study are denoted with strain codes and corresponding GenBank accession numbers (PX149150-PX149154). *Saccharomyces cerevisiae* was used as an outgroup to root the tree
2. **Supplementary Figure 2.** Effect of bacterial treatments (Ck, *S*. *jiujiangensis* NA-06, *B. cepacia* NA-07, *P. dispersa* NA-08, *Pseudomonas* sp. NA-15, *V. guangxiensis* NA-16, Consortium-I, Consortium-II and Consortium-III) on shoot length (SL) of drought susceptible (Ranjit) and tolerant (CR Dhan 802) rice varieties under non-stress and drought conditions. Here, (a) CR Dhan 802 and (b) Ranjit. Bars represent mean ± standard deviation (SD) of three biological replicates (n = 3). Different letters above bars indicate pairwise significant differences among treatment groups within each condition as determined by Tukey's HSD post hoc test
3. **Supplementary Figure 3.** Effect of bacterial treatments (Ck, *S*. *jiujiangensis* NA-06, *B. cepacia* NA-07, *P. dispersa* NA-08, *Pseudomonas* sp. NA-15, *V. guangxiensis* NA-16, Consortium-I, Consortium-II and Consortium-III) on shoot dry weight (SDW), root dry weight (RDW), and relative water content (RWC) of drought susceptible (Ranjit) and tolerant (CR Dhan 802) rice varieties under non-stress and drought conditions. Here, (a-b) SDW, (c-d) RDW, and (e-f) RWC of CR Dhan 802 and Ranjit, respectively. Bars represent mean ± standard deviation (SD) of three biological replicates (n = 3). Different letters above bars indicate pairwise significant differences among treatment groups within each condition as determined by Tukey's HSD post hoc test

**Supplementary Figure 1.** Phylogenetic tree illustrating the evolutionary relationships of the selected rice rhizospheric bacterial isolates based on 16S rRNA gene sequences. The tree was constructed using the Maximum likelihood method with 1000 bootstrap replications. Bootstrap values (>30%) are indicated at the branch nodes. The isolates obtained in this study are denoted with strain codes and corresponding GenBank accession numbers (PX149150-PX149154). Saccharomyces cerevisiae was used as an outgroup to root the tree

**Supplementary Figure 2.** Effect of bacterial treatments (Ck, *S*. *jiujiangensis* NA-06, *B. cepacia* NA-07, *P. dispersa* NA-08, *Pseudomonas* sp. NA-15, *V. guangxiensis* NA-16, Consortium-I, Consortium-II and Consortium-III) on shoot length (SL) of drought susceptible (Ranjit) and tolerant (CR Dhan 802) rice varieties under non-stress and drought conditions. Here, (a) CR Dhan 802 and (b) Ranjit. Bars represent mean ± standard deviation (SD) of three biological replicates (n = 3). Different letters above bars indicate pairwise significant differences among treatment groups within each condition as determined by Tukey's HSD post hoc test

**Supplementary Figure 3.** Effect of bacterial treatments (Ck, *S*. *jiujiangensis* NA-06, *B. cepacia* NA-07, *P. dispersa* NA-08, *Pseudomonas* sp. NA-15, *V. guangxiensis* NA-16, Consortium-I, Consortium-II and Consortium-III) on shoot dry weight (SDW), root dry weight (RDW), and relative water content (RWC) of drought susceptible (Ranjit) and tolerant (CR Dhan 802) rice varieties under non-stress and drought conditions. Here, (a-b) SDW, (c-d) RDW, and (e-f) RWC of CR Dhan 802 and Ranjit, respectively. Bars represent mean ± standard deviation (SD) of three biological replicates (n = 3). Different letters above bars indicate pairwise significant differences among treatment groups within each condition as determined by Tukey's HSD post hoc test
