# Supplementary material for: Global meta-analysis of drought associated rice rhizosphere microbiome data and soil application of selected bacteria reveals potential of Pantoea dispersa for drought stress alleviation in rice plants
Source: Front Plant Sci. 2026 Sep 3;17:1904089. doi: 10.3389/fpls.2026.1904089 (PMC13581755; doi:10.3389/fpls.2026.1904089)
Supplement: Supplementary file 2 [file SupplementaryFile2.docx]

**Supplementary Information**

**List of Tables:**

1. **Supplementary Table 1** Overview of samples metadata and high-throughput sequencing information, including primer sets and sequencing platforms
2. **Supplementary Table 2** Overview of samples metadata and high-throughput sequencing information, including primer sets and sequencing platforms
3. **Supplementary Table 3** Differential abundance analysis of bacterial communities in rice rhizosphere under control and drought conditions
4. **Supplementary Table 4** Plant growth-promoting traits of selected drought-associated rhizospheric bacterial isolates (n=3)
5. **Supplementary Table 5** Optical density (OD₆₀₀) of bacterial isolates under control and PEG treatments at 24 and 96 h of incubation
6. **Supplementary Table 6** Comparative analysis of plant physiological, biochemical and antioxidant responses of CR Dhan 802 with bacterial inoculation under non-stress and drought conditions
7. **Supplementary Table 7** Two-way ANOVA F-values and probability (p) for physiological, biochemical, and antioxidant responses of CR Dhan 802 with bacterial inoculation under non-stress and drought conditions, with Tukey HSD post-hoc comparisons
8. **Supplementary Table 8** Comparative analysis of plant physiological, biochemical and antioxidant responses of Ranjit with bacterial inoculation under non-stress and drought conditions
9. **Supplementary Table 9** Two-way ANOVA F-values and probability (p) for physiological, biochemical, and antioxidant responses of Ranjit with bacterial inoculation under non-stress and drought conditions, with Tukey HSD post-hoc comparisons
10. **Supplementary Table 10** Comparative analysis soil enzymatic activities of CR Dhan 802 and Ranjit with bacterial inoculation under non-stress and drought conditions
11. **Supplementary Table 11** Two-way ANOVA F-values and probability (p) for soil enzymatic activities of CR Dhan 802 and Ranjit with bacterial inoculation under non-stress and drought conditions, with Tukey HSD post-hoc comparisons

**Supplementary Table 1** Overview of samples metadata and high-throughput sequencing information, including primer sets and sequencing platforms

| **Accession No.** | **Seq_line** | **Country** | **Primers** | **Region** | **Zone** | **Host/plant** | **References** |
| --- | --- | --- | --- | --- | --- | --- | --- |
| PRJNA631648 | Illumina HiSeq | China | 515F/806R | V4 | Rhizosphere | *Oryza longistaminata* | Xie et al., 2021 |
| PRJNA551661 | Illumina MiSeq | USA | 515F/806R | V4 | Rhizosphere | *O. sativa* subsp. *japonica* cv. M-206 | Santos-Medellín et al., 2021 |
| CRA005699 | Illumina NovaSeq | China | 343F/798R | V3–V4 | Rhizosphere | *O. sativa L. subsp. indica* cv*. Huanghuazhan* | Zhu et al., 2022 |
| CRA005709 | Illumina MiSeq | China | 343F/798R | V3–V4 | Rhizosphere | *O. sativa L. subsp. japonica* cv. *Yangda 3* | Sun et al., 2023 |
| PRJNA907252 | Illumina MiSeq | China | 338F/806R | V3–V4 | Rhizosphere | *O. sativa* L. subsp. *japonica* cv. Nipponbare, *O. sativa* L. cv. Luodao 998 | Li et al., 2023 |
| PRJNA739870 | Illumina MiSeq | China | 799F/119R | V5–V7 | Rhizosphere and bulk soil | *O. sativa* L. subsp. *japonica* cv. Nipponbare | Xu et al., 2022 |
| PRJNA386367 | Illumina Miseq | US | 515F/806R | V4 | Rhizosphere and bulk soil | *O. sativa*-M206 (subsp. *japonica*) and IR20 (subsp. *indica*); *Oryza* *glaberrima-* TOg7102 and CG14 | Santos-Medellín et al., 2017 |
| PRJNA944153 | Illumina MiSeq | China | 338F/806R | V3–V4 | Rhizosphere and bulk soil | O. sativa L. subsp. japonica cv. Dongjing | Lei et al., 2023 |
| PRJNA900492 | Illumina MiSeq | India | 341F/805R | V3–V4 | Rhizosphere and bulk soil | *O. sativa* L. cv. Norungan, *O. sativa* L. cv. Co51 | Nunna and Balachandar, 2024 |
| CRA012577 | Illumina MiSeq | China | 322F-A/796R | V3–V4 | Rhizosphere and bulk soil | O.sativa L. subsp. japonica cv. Jia 67 | Wu et al., 2024 |
| SRP057189 | Illumina MiSeq | Italy | 515F/806R | V4 | Bulk soil | - | Breidenbach et al., 2016 |
| PRJNA736197 | Illumina MiSeq | Canada | 520F/799R | V4 | Bulk soil | - | Azarbad et al., 2022 |
| PRJNA544797 | Illumina MiSeq | USA | 515F/806R | V4 | Bulk soil | - | Wang et al., 2020 |
| PRJNA943479 | Illumina MiSeq | China | 515F/806R | V4 | Bulk soil | - | Fang et al., 2023 |

**Supplementary Table 2** Overview of samples metadata and high-throughput sequencing information, including primer sets and sequencing platforms

| **Bacterial Genera** | **Significance under drought** | **Studied crop plants** | **References** |
| --- | --- | --- | --- |
| *Streptomyces* | *S. coelicolor* Sc1 and *S. ambofaciens* Sc2 enriched under drought conditions; associated with drought-induced selection in the root microbiome | sorghum (*Sorghum bicolor* (L.) Moench) | Xu et al., 2018 |
|  | *S. pactum* Act12 enhanced drought resistance by enhancing plant anti-oxidative enzymatic activities and up regulate the expression of drought responsive genes such as *EXPA2*, *EXPA6*, *P5CS*, and *SnRK2* | drought sensitive wheat (*Triticum aestivum* L.) cultivar Xinong 979 | Li et al., 2020 |
|  | *S*. *chartreusis* WZS021 enhances drought tolerance by improving rhizosphere nutrient status, plant anti-oxidant enzyme activities, and by regulating plant hormonal pathways through signalling molecules such as [petunidin](https://www.sciencedirect.com/topics/biochemistry-genetics-and-molecular-biology/petunidin), salicylic acid, α-Linoleic acid, auxin, [geranylgeraniol](https://www.sciencedirect.com/topics/biochemistry-genetics-and-molecular-biology/geranylgeraniol) and [phenylalanine](https://www.sciencedirect.com/topics/biochemistry-genetics-and-molecular-biology/phenylalanine) and key genes including *YUCCA, amiE, AUX, CYPs,* and *PAL.* | sugarcane (*Saccharum officinarum* L.) | Pang et al., 2024 |
|  | Drought-induced accumulation of 4-oxoproline promotes enrichment of *S. coeruleorubidus* in the rhizosphere, increases expression of the bacterial drought-responsive gene (*rimJ*); and re-inoculation improves host drought tolerance | wheat (*Triticum aestivum* L.) | Li, J. et al., 2025 |
|  | *Streptomyces* sp. N2A alleviates drought stress by promoting root development, maintaining photosynthesis, improving water-use efficiency, and enhancing plant physiological responses, thereby reducing yield loss. | soybean (*Glycine max* (L.) Merr.) | Villafane et al., 2024 |
| *Burkholderia* | Co-inoculation of *B. phytofirmans* PsJN and *Enterobacter* sp. FD17 enhanced drought resilience via increasing water-use efficiency, chlorophyll retention, photosynthetic pigment stability, and reduced drought-induced oxidative damage. | maize (*Zea mays* L.) | Naveed et al., 2014 |
|  | B. phytofirmans PsJN modulate host plant genes involved in transcriptional regulation, cellular homeostasis, and ROS detoxification, including extracytoplasmic function (ECF) sigma factor genes. | potato (*Solanum tuberosum* L**.**) | Sheibani-Tezerji et al., 2015 |
|  | *B. seminalis* in combination with *Rhizophagus* spp. improved plant growth and drought tolerance by enhancing nutrient uptake, root development, and biomass accumulation. | tomato (*Lycopersicon esculatum*) and bell pepper (*Capsicum annuum*) | Tallapragada et al., 2016 |
|  | Inoculation of *Burkholderia* sp. reduce soil water loss, enhanced plant proline and MDA levels, anti-oxidative enzymes, and phytohormones | *Arabidopsis thaliana* and maize (*Z. mays* L.) | Huang et al., 2017 |
| *Pantoea* | *P. alhagi* improved drought tolerance by enhancing root growth, increasing chlorophyll content, promoting osmolyte accumulation, improving antioxidant enzyme activities, and reducing oxidative damage. | wheat (*T. aestivum* L.) | Chen et al., 2017 |
|  | *P. agglomerans* induced drought tolerance through enhancement of antioxidant capacity, osmoprotection, phytohormone-related functions, and secondary metabolite biosynthesis. | durum wheat (*Triticum turgidum* ssp. *durum*) | Cherif-Silini et al., 2019 |
|  | Exopolysaccharide derived from *P. alhagi* NX-11 enhanced rice drought resistance by improving water retention capacity, antioxidant defense, osmotic balance, and stress-responsive physiological traits. | rice (*Oryza sativa* L.) | Sun et al., 2020 |
|  | *P. agglomerans* BCH-1, improved plant biomass, photosynthetic pigments, osmolyte accumulation, antioxidant defense, and nutrient uptake. It also produces volatile organic compounds (VOCs) such as tridecane, 1-hexadecene, 1-octadecene, tetradecane, pyrrolo[1,2-a] pyrazine-1,4-dione, hexahydro-3-(2-methylpropyl)-, 1- docosene, hexadecanoic acid, methyl ester, and tetradecanoic acid that activate plant metabolic and defense pathways under drought | maize (*Z. mays* L.) | Tariq et al., 2025 |
| *Pseudomonas* | Plant growth-promoting *Pseudomonas* spp. improved maize drought tolerance by increasing solute accumulation, and enhancing anti-oxidant enzymatic activities | maize (*Z. mays* L.) | Sandhya et al., 2010 |
|  | P. aeruginosa GGRJ21 alleviated drought stress by increasing plant biomass, water use efficacy, and upregulating drought responsive genes such as *DREB2A, CAT1* and *DHA.* | mung bean (*Vigna radiata* (L.) R. Wilczek) | Sarma and Saikia, 2014 |
|  | P. putida enhances drought tolerance by upregulating genes involved in transcriptional regulation (*DREB1A*, *NAC1*), stress response (*LEA*, *DHN*), ROS scavenging (*CAT*, *APX*, *GST*), and hormone signaling pathways including ethylene (*ACO*, *ACS*), salicylic acid (*PR1*), and jasmonate (*MYC2)* in both the cultivars. | chickpea (Cicer arietinum L.) cultivars cv. BG-362 and cv. BG-1003 | Tiwari et al., 2016 |
|  | *Pseudomonas* strains (PS1, PS2, PS3, and PS4) isolated from the rice and maize rhizosphere under different water regimes, showed plant drought tolerance by promoting plant water use efficacy, osmolytes accumulation, and reduces oxidative damage through accumulation of antioxidants | *A. thaliana* | Yasmin et al., 2022 |
| *Variovorax* | *V. paradoxus* 5C-2 promotes growth via modulating ethylene dependent pathway and restores root and shoot growth | *A. thaliana* | Chen et al., 2013 |
|  | *V. paradoxus* RAA3 improve plant growth; significantly upregulating the expression of the drought-responsive aquaporin gene *TaTIP1;1* and *DEAD-box helicase gene WDH45* in drought-tolerant variety PBW 660 | wheat (*Triticum aestivum* L.) drought-tolerant variety PBW 660, drought-sensitive variety HD 2967 | Chandra et al., 2019 |
|  | Variovorax sp. YNA59 enhanced drought tolerance by improving plant growth, modulating phytohormone balance, increasing antioxidant defense, promoting osmolyte accumulation | broccoli (*Brassica oleracea* var. *italica* L.) | Kim et al., 2020 |
|  | Variovorax spp. were identified as a core drought-associated genus in the sorghum rhizosphere, and their presence is associated with improved plant performance under drought stress conditions. | sorghum (*Sorghum bicolor* (L.) Moench) | Qi et al., 2022 |

**Supplementary Table 3** Differential abundance analysis of bacterial communities in rice rhizosphere under control and drought conditions

| **Taxon_Genus** | **Conditions** | **LDA score** | **P.adj** | **P.unadj** | **Phylum** | **Class** | **Order** | **Family** |
| --- | --- | --- | --- | --- | --- | --- | --- | --- |
| *Anaeromyxobacter* | Control | 4.2208 | 2.43951E-11 | 6.59328E-13 | Myxococcota | Myxococcia | Myxococcales | Anaeromyxobacteraceae |
| *Denitratisoma* | Control | 3.8718 | 2.43951E-11 | 6.20729E-13 | Pseudomonadota | Gammaproteobacteria | Burkholderiales | Rhodocyclaceae |
| *Candidatus Koribacter* | Control | 3.6620 | 4.16424E-13 | 2.81368E-15 | Acidobacteriota | Acidobacteriae | Terriglobales | Koribacteraceae |
| *Streptomyces* | Drought | 3.5881 | 2.60672E-12 | 3.52259E-14 | Actinomycetota | Actinobacteria | Kitasatosporales | Streptomycetaceae |
| *Marmoricola* | Drought | 3.2071 | 8.25291E-07 | 3.90340E-08 | Actinomycetota | Actinobacteria | Propionibacteriales | Nocardioidaceae |
| *Lysobacter* | Drought | 3.0625 | 0.00161948 | 0.000164238 | Pseudomonadota | Gammaproteobacteria | Lysobacterales | Lysobacteraceae |
| *Novosphingobium* | Drought | 2.9987 | 0.006576828 | 0.001155389 | Pseudomonadota | Alphaproteobacteria | Sphingomonadales | Sphingomonadaceae |
| possible genus 04 | Drought | 2.9545 | 4.76883E-09 | 1.61109E-10 | Fibrobacterota | Fibrobacteria | Fibrobacterales | Fibrobacteraceae |
| *Blastococcus* | Drought | 2.9314 | 0.001729989 | 0.000222093 | Actinomycetota | Actinobacteria | Frankiales | Geodermatophilaceae |
| *Luteitalea* | Drought | 2.8981 | 0.001487259 | 0.000130638 | Acidobacteriota | Vicinamibacteria | Vicinamibacterales | Vicinamibacteraceae |
| *Brevibacterium* | Drought | 2.8626 | 5.94996E-05 | 3.61822E-06 | Actinomycetota | Actinobacteria | Micrococcales | Brevibacteriaceae |
| *Priestia* | Drought | 2.8462 | 0.00161948 | 0.000190643 | Bacillota | Bacilli | Bacillales | Bacillaceae |
| *Bradyrhizobium* | Drought | 2.8444 | 0.000724423 | 5.38422E-05 | Pseudomonadota | Alphaproteobacteria | Hyphomicrobiales | Xanthobacteraceae |
| *Mycobacterium* | Drought | 2.7226 | 0.00461035 | 0.000716473 | Actinomycetota | Actinobacteria | Mycobacteriales | Mycobacteriaceae |
| *Amycolatopsis* | Drought | 2.6552 | 0.000255449 | 1.72601E-05 | Actinomycetota | Actinobacteria | Pseudonocardiales | Pseudonocardiaceae |

**Supplementary Table 4** Plant growth-promoting traits of selected drought-associated rhizospheric bacterial isolates (n=3)

| **Bacterial isolates** | **ACC Deaminase Activity**  **(µM α-ketobutyrate mg⁻¹h⁻¹)** | **IAA production**  **(µg ml^-1^)** | **Phosphate Solubilisation Index (PSI)** |
| --- | --- | --- | --- |
| *S. jiujiangensis* NA-06 | 13.35±3.96 | 32.58±1.71 | 2.49±0.13 |
| *B. cepacia* NA-07 | 18.15±2.83 | 27.59±3.74 | 3.34±0.17 |
| *P. dispersa* NA-08 | 35.08±1.72 | 31.2±0.97 | 3.31±0.13 |
| *Pseudomonas* sp. NA-15 | 1.85±2.05 | 9.61±1.62 | 2.8±0.15 |
| *V. guangxiensis* NA-16 | 20.23±5.18 | 34.50±3.75 | 1.89±0.13 |

**Supplementary Table 5** Optical density (OD₆₀₀) of bacterial isolates under control and PEG treatments at 24 and 96 hrs of incubation

| **Isolate** | **Treatment** | **24 h OD₆₀₀** | **96 h OD₆₀₀** |
| --- | --- | --- | --- |
| *S. jiujiangensis* NA-06 | Control | 0.00±0.00^h^ | 0.357 ± 0.010^fg^ |
|  | PEG | 0.00±0.00^h^ | 0.140 ± 0.029^g^ |
| *B. cepacia* NA-07 | Control | 1.547 ± 0.042^b^ | 2.345 ± 0.255^b^ |
|  | PEG | 0.323 ± 0.007^f^ | 0.839 ± 0.026^d^ |
| *P. dispersa* NA-08 | Control | 1.062 ± 0.019^d^ | 1.489 ± 0.040^c^ |
|  | PEG | 0.452 ± 0.038^e^ | 0.764 ± 0.013^de^ |
| *Pseudomonas* sp. NA-15 | Control | 1.733 ± 0.101^a^ | 2.920 ± 0.246^a^ |
|  | PEG | 0.173 ± 0.006^g^ | 0.521 ± 0.011^def^ |
| *V. guangxiensis* NA-16 | Control | 1.372 ± 0.045^c^ | 2.054 ± 0.064^b^ |
|  | PEG | 0.247 ± 0.005^fg^ | 0.461 ± 0.006^efg^ |

Values are presented as mean ± SD (n = 3). Different superscript letters within a column indicate significant differences among treatments according to Tukey's HSD test (p < 0.05).

**Supplementary Table 6** Comparative analysis of plant physiological, biochemical and antioxidant responses of CR Dhan 802 with bacterial inoculation under non-stress and drought conditions

| **Treatments** | **Conditions** | **SL**  **(cm)** | **RWC %** | **RDW**  **(g plant^-1^)** | **SDW**  **(g plant^-1^)** | **TC**  **(mg g ^-1^ FW)** | **CAR**  **(mg g ^-1^ FW)** | **TPC**  **(mg GAE g^-1^ FW)** | **PRO**  **(µmol g⁻¹ FW)** | **SOD**  **(U min ^-1^ g^-1^ FW)** | **CAT**  **(U min ^-1^ g^-1^ FW)** | **H_2_O_2_**  **(µ mol g^-1^ FW)** | **MDA**  **(µ mol g^-1^ FW)** |
| --- | --- | --- | --- | --- | --- | --- | --- | --- | --- | --- | --- | --- | --- |
| NT-Ck | Non-stress | 48.76 ± 1.04ᵇᶜᵈ | 76.80±1.85ᵇᶜᵈ | 0.304±0.007ʰⁱʲ | 1.270±0.02ᵍ | 6.87±0.1ᵃ^b^ | 1.17±0.02^abc^ | 3.21±0.07ⁱ | 36.83±4.31ᵉ | 162.23±6.39ˡ | 122.70±8.52ᵉ | 0.94±0.02ᶠᵍ | 0.029±0.000ᶠ |
|  | Drought | 46.40 ± 0.55ᵈ | 34.90±3.61ʰ | 0.273±0.006ᵏ | 0.734±0.02ᵐ | 1.2±0.05^g^ | 0.23±0.01^h^ | 3.81±0.07ᵉᶠᵍ | 76.93±4.12ᵈ | 294.60±6.44ᵉ | 198.97±6.26ᵈ | 2.30±0.03ᵃ | 0.053±0.001ᵃ |
| *Streptomyces jiujiangensis* NA-06 | Non-stress | 52.87 ± 5.45ᵃᵇᶜᵈ | 84.57±1.88ᵃᵇ | 0.346±0.007ᵃᵇᶜ | 1.438±0.02ᵇᶜ | 7.08±0.90ᵃ^b^ | 1.21±0.15^ab^ | 3.76±0.06ᶠᵍ | 39.33±3.01ᵉ | 234.77±5.50ᵍ | 125.10±3.66ᵉ | 0.77±0.02ʲᵏ | 0.018±0.000ⁱ |
|  | Drought | 50.46 ± 4.71ᵇᶜᵈ | 64.97±3.06ᵉ | 0.317±0.008ᵈᵉᶠᵍʰ | 0.918±0.02ᶦʲ | 1.19±0.03^g^ | 0.22±0.01^h^ | 4.56±0.06ᵇ | 87.20±4.19ᵇᶜᵈ | 382.20±6.42ᵇ | 218.50±6.10ᵇᶜ | 1.82±0.02ᵈ | 0.039±0.001ᵈ |
| *Burkholderia cepacia* NA-07 | Non-stress | 57.52 ± 4.15ᵃᵇ | 87.63±1.75ᵃ | 0.355±0.008ᵃᵇ | 1.462±0.03ᵇ | 5.69±0.13^cd^ | 1±0.02^cde^ | 3.96±0.06ᵈᵉ | 42.17±2.70ᵉ | 261.27±5.58ᶠ | 132.10±3.80ᵉ | 0.74±0.02ᵏˡ | 0.015±0.000ʲ |
|  | Drought | 55.86 ± 3.49ᵃᵇᶜ | 72.63±3.05ᵈᵉ | 0.327±0.008ᶜᵈᵉᶠᵍ | 0.972±0.02ʰᶦ | 1.16±10.03^g^ | 0.22±0.01^h^ | 4.91±0.07ᵃ | 98.70±4.32ᵃ | 422.27±5.83ᵃ | 259.00±7.35ᵃ | 1.56±0.02ᵉ | 0.034±0.000ᵉ |
| *Pantoea dispersa* NA-08 | Non-stress | 53.37 ± 3.23ᵃᵇᶜᵈ | 88.37±1.82ᵃ | 0.338±0.008ᵇᶜᵈ | 1.412±0.02ᵇᶜᵈ | 5.43±0.08^cd^ | 0.95±0.01^de^ | 3.71±0.07ᶠᵍ | 42.97±2.76ᵉ | 262.87±5.64ᶠ | 133.43±4.02ᵉ | 0.81±0.02ⁱʲ | 0.013±0.001ʲ |
|  | Drought | 51.67 ± 1.13ᵇᶜᵈ | 73.50±3.12ᵈ | 0.334±0.008ᵇᶜᵈᵉ | 1.008±0.02ʰ | 1.17±0.03^g^ | 0.22±0.01^h^ | 4.93±0.06ᵃ | 100.50±4.56ᵃ | 424.87±6.31ᵃ | 261.73±7.16ᵃ | 1.55±0.02ᵉ | 0.034±0.001ᵉ |
| *Pseudomonas* sp. NA-15 | Non-stress | 53.63 ± 0.42ᵃᵇᶜ^d^ | 76.43±1.82ᶜᵈ | 0.321±0.007ᵈᵉᶠᵍʰ | 1.354±0.02ᵈᵉᶠ | 2.53±0.49^f^ | 0.46±0.09ᵍ | 3.46±0.06ʰ | 37.87±2.86ᵉ | 169.93±5.52ᵏˡ | 122.80±3.70ᵉ | 0.89±0.02ᵍʰ | 0.025±0.002ᵍ |
|  | Drought | 50.09 ± 1.54ᵇᶜᵈ | 35.93±3.16ʰ | 0.286±0.007ʲᵏ | 0.768±0.02ˡᵐ | 1.13±0.07^g^ | 0.21±0.01^h^ | 3.83±0.06ᵉᶠᵍ | 78.47±4.41ᶜᵈ | 296.10±6.40ᵉ | 201.93±6.42ᶜᵈ | 2.31±0.03ᵃ | 0.052±0.001ᵃ |
| *Variovorax guangxiensis* NA-16 | Non-stress | 60.98 ± 3.54ᵃ | 81.90±1.11ᵃᵇᶜ | 0.365±0.009ᵃ | 1.565±0.04ᵃ | 7.78±0.33ᵃ | 1.32±0.06^a^ | 3.86±0.06ᵉᶠ | 39.47±3.12ᵉ | 208.37±5.33ʰ | 125.80±3.60ᵉ | 0.69±0.02ˡ | 0.018±0.000ⁱ |
|  | Drought | 52.53 ± 4.83ᵃᵇᶜᵈ | 55.97±2.58ᶠ | 0.309±0.008ᶠᵍʰⁱ | 0.856±0.02ʲᵏ | 1.22±0.03^g^ | 0.23±0.01^h^ | 4.28±0.07ᶜ | 89.27±4.24ᵃᵇᶜ | 351.90±6.20ᶜ | 219.60±6.20ᵇ | 1.96±0.03ᶜ | 0.044±0.001ᶜ |
| Consortium-I | Non-stress | 57.08 ± 1.80ᵃᵇᶜ | 81.77±1.88ᵃᵇᶜ | 0.330±0.007ᶜᵈᵉᶠ | 1.386±0.02ᶜᵈᵉ | 4.21±0.47^e^ | 0.75±0.08ᶠ | 3.66±0.06ᵍ | 41.00±2.96ᵉ | 207.50±5.66ʰᶦ | 127.70±3.63ᵉ | 0.84±0.02ʰᶦ | 0.021±0.000ʰ |
|  | Drought | 54.51 ± 2.44ᵃᵇᶜᵈ | 55.60±3.16ᶠ | 0.301±0.008ʰᶦʲ | 0.894±0.02ʲ | 1.13±0.02^g^ | 0.21±0.004^h^ | 4.31±0.07ᶜ | 91.90±4.19ᵃᵇ | 350.30±6.14ᶜ | 224.37±5.77ᵇ | 1.97±0.02ᶜ | 0.043±0.001ᶜ |
| Consortium-II | Non-stress | 55.81 ± 2.41ᵃᵇᶜ | 78.90±2.17ᵇᶜᵈ | 0.307±0.006ᵍʰⁱʲ | 1.302±0.02ᶠᵍ | 6.11±0.01^bc^ | 1.05±0.02^bcd^ | 3.26±0.06ᶦ | 38.83±2.94ᵉ | 187.70±5.55ʲᵏ | 124.53±3.20ᵉ | 0.96±0.02ᶠ | 0.022±0.000ʰ |
|  | Drought | 54.36 ± 1.29ᵃᵇᶜᵈ | 44.43±3.42ᵍ | 0.279±0.007ᵏ | 0.784±0.02ˡᵐ | 1.17±0.03^g^ | 0.23±0.01^h^ | 4.06±0.06ᵈ | 83.33±4.15ᵇᶜᵈ | 321.63±6.15ᵈ | 207.67±6.15ᵇᶜᵈ | 2.17±0.02ᵇ | 0.038±0.000ᵈ |
| Consortium-III | Non-stress | 50.42 ± 1.26ᵇᶜᵈ | 78.97±1.51ᵇᶜᵈ | 0.314±0.006ᵉᶠᵍʰⁱ | 1.328±0.02ᵉᶠᵍ | 4.80±0.8^de^ | 0.85±0.14^ef^ | 3.36±0.06ʰⁱ | 39.33±2.94ᵉ | 190.07±5.68ⁱʲ | 125.47±3.48ᵉ | 0.92±0.02ᶠᵍ | 0.025±0.000ᵍ |
|  | Drought | 48.40 ± 2.57ᶜᵈ | 45.03±3.29ᵍ | 0.293±0.007ᶦʲᵏ | 0.812±0.02ᵏˡ | 1.14±0.03^g^ | 0.21±0.01^h^ | 4.08±0.07ᵈ | 84.63±4.05ᵇᶜᵈ | 324.33±5.76ᵈ | 209.47±6.15ᵇᶜᵈ | 2.20±0.02ᵇ | 0.047±0.001ᵇ |

Values are presented as mean ± standard deviation (SD) of three biological replicates (n = 3). Data were analyzed using two-way analysis of variance (ANOVA) to evaluate the effects of bacterial treatments on CR Dhan 802 under control vs. drought conditions, and their interaction. Mean comparisons were performed using Tukey’s honestly significant difference (HSD) test. Different lowercase letters indicate statistically significant differences among treatments. Here, SL- shoot length**, RWC- relative water content, RDW- root dry weight, SDW- shoot dry weight, TC- total chlorophyll content, CAR- carotenoid content, TPC- total phenol content, PRO-proline content, SOD-Superoxide dismutase, CAT- catalase activity, H_2_O_2_- hydrogen peroxide content, MDA- malondialdehyde content.**

**Supplementary Table 7** Two-way ANOVA F-values and probability (p) for physiological, biochemical, and antioxidant responses of CR Dhan 802 with bacterial inoculation under non-stress and drought conditions, with Tukey HSD post-hoc comparisons

| **Effects** | **df** | **SL (cm)** | | | | **RWC (%)** | | | | **RDW (g plant⁻¹)** | | | |
| --- | --- | --- | --- | --- | --- | --- | --- | --- | --- | --- | --- | --- | --- |
|  |  | **Sum Sq.** | **Mean Sq.** | **F value** | **p value** | **Sum Sq.** | **Mean Sq.** | **F value** | **p value** | **Sum Sq.** | **Mean Sq.** | **F value** | **p value** |
| Conditions (C) | **1** | 114.0954 | 114.0954 | 13.0251 | < 0.001^***^ | 10614.26 | 10614.26 | 1605.3234 | < 0.001^***^ | 0.01141 | 0.01141 | 226.8870 | < 0.001^***^ |
| Treatments (T) | **8** | 512.5656 | 64.0707 | 7.3143 | < 0.001^***^ | 4344.4767 | 543.0596 | 82.1335 | < 0.001^***^ | 0.01968 | 0.002460 | 48.9093 | < 0.001^***^ |
| C × T | **8** | 56.6576 | 7.0822 | 0.80851 | 0.5996^ns^ | 1268.9388 | 158.6173 | 23.9896 | < 0.001^***^ | 0.002171 | 0.000271 | 5.3953 | < 0.001^***^ |
| Residuals | **36** | 315.3465 | 8.7596 | — | — | 238.0289 | 6.6119 | — | — | 0.001811 | 5.0296e-5 | — | — |
|  | | | | | | | | | | | | | |
| **Effects** | **df** | **SDW (g plant⁻¹)** | | | | **TC (mg g⁻¹ FW)** | | | | **CAR (mg g⁻¹ FW)** | | | |
|  |  | **Sum Sq.** | **Mean Sq.** | **F value** | **p value** | **Sum Sq.** | **Mean Sq.** | **F value** | **p value** | **Sum Sq.** | **Mean Sq.** | **F value** | **p value** |
| Conditions (C) | **1** | 3.79215 | 3.79215 | 8921.93 | < 0.001^***^ | 266.63 | 266.63 | 2328.47 | < 0.001^***^ | 7.67 | 7.67 | 2313.42 | < 0.001^***^ |
| Treatments (T) | **8** | 0.3299 | 0.04123 | 97.021741 | < 0.001^***^ | 32.67 | 4.08 | 35.66 | < 0.001^***^ | 0.87 | 0.11 | 32.89 | < 0.001^***^ |
| C × T | **8** | 0.08178 | 0.01022 | 24.05065 | < 0.001^***^ | 30.26 | 3.78 | 33.03 | < 0.001^***^ | 0.80 | 0.1 | 30.04 | < 0.001^***^ |
| Residuals | **36** | 0.0153 | 0.00043 | — | — | 4.12 | 0.11 | — | — | 0.12 | 0.003 | — | — |
|  | | | | | | | | | | | | | |
| **Effects** | **df** | **TPC (mg GAE g⁻¹ FW)** | | | | **PRO (µmol g⁻¹ FW)** | | | | **SOD (U min⁻¹ g⁻¹ FW)** | | | |
|  |  | **Sum Sq.** | **Mean Sq.** | **F value** | **p value** | **Sum Sq.** | **Mean Sq.** | **F value** | **p value** | **Sum Sq.** | **Mean Sq.** | **F value** | **p value** |
| Conditions (C) | **1** | 7.1723 | 7.1723 | 1840.7909 | < 0.001^***^ | 31267.41 | 31267.41 | 2259.3574 | < 0.001^***^ | 274562.04 | 274562.04 | 7814.9001 | < 0.001^***^ |
| Treatments (T) | **8** | 5.1313 | 0.64142 | 164.6223 | < 0.001^***^ | 1228.9148 | 153.6144 | 11.1000 | < 0.001^***^ | 88391.22 | 11048.90 | 314.4865 | < 0.001^***^ |
| C × T | **8** | 0.81403 | 0.10175 | 26.1156 | < 0.001^***^ | 467.9059 | 58.4882 | 4.2263 | < 0.01^**^ | 1887.7833 | 235.9729 | 6.7165 | < 0.001^***^ |
| Residuals | **36** | 0.14027 | 0.003896 | — | — | 498.2067 | 13.8391 | — | — | 1264.7933 | 35.1331 | — | — |
|  | | | | | | | | | | | | | |
| **Effects** | **df** | **CAT (U min⁻¹ g⁻¹ FW)** | | | | **H₂O₂ (µmol g⁻¹ FW)** | | | | **MDA (µmol g⁻¹ FW)** | | | |
|  |  | **Sum Sq.** | **Mean Sq.** | **F value** | **p value** | **Sum Sq.** | **Mean Sq.** | **F value** | **p value** | **Sum Sq.** | **Mean Sq.** | **F value** | **p value** |
| Conditions (C) | **1** | 123724.80 | 123724.80 | 4056.0069 | < 0.001^***^ | 17.5446 | 17.5446 | 49344.19 | < 0.001^***^ | 0.006660 | 0.006660 | 11988.92 | < 0.001^***^ |
| Treatments (T) | **8** | 8656.8072 | 1082.1009 | 35.4740 | < 0.001^***^ | 1.6065 | 0.20081 | 564.7708 | < 0.001^***^ | 0.001716 | 0.000215 | 386.2258 | < 0.001^***^ |
| C × T | **8** | 4489.6113 | 561.2014 | 18.3976 | < 0.001^***^ | 0.66087 | 0.08261 | 232.3359 | < 0.001^***^ | 0.000137 | 1.7177e-5 | 30.9197 | < 0.001^***^ |
| Residuals | **36** | 1098.1473 | 30.5041 | — | — | 0.01280 | 0.000356 | — | — | 1.9999e-5 | 5.5553e-7 | — | — |

Here, *** p < 0.001 (highly significant); ** p < 0.01 (highly significant); * p < 0.05 (significant); ns = not significant

**Supplementary Table 8** Comparative analysis of plant physiological, biochemical and antioxidant responses of Ranjit with bacterial inoculation under non-stress and drought conditions

| **Treatments** | **Conditions** | **SL**  **(cm)** | **RWC %** | **RDW**  **(g plant^-1^)** | **SDW**  **(g plant^-1^)** | **TC**  **(mg g ^-1^ FW)** | **CAR**  **(mg g ^-1^ FW)** | **TPC**  **(mg GAE g^-1^ FW)** | **PRO**  **(µmol g⁻¹ FW)** | **SOD**  **(U min ^-1^ g^-1^ FW)** | **CAT**  **(U min ^-1^ g^-1^ FW)** | **H_2_O_2_**  **(µ mol g^-1^ FW)** | **MDA**  **(µ mol g^-1^ FW)** |
| --- | --- | --- | --- | --- | --- | --- | --- | --- | --- | --- | --- | --- | --- |
| NT-Ck | Non-stress | 53.08 ± 4.5ᵃᵇᶜ | 64.157±3.622ᵃ | 0.165±0.005ᵍʰⁱʲ | 1.085±0.016ᵍ | 1.58±0.03ᵃ | 0.29±0.01^a^ | 2.11±0.07ˡ | 36.67±5.35ᵈ | 128.37±6.79ⁱ | 91.14±3.70ᶠ | 1.15±0.03ᶠ | 0.045±0.000ᵍ |
|  | Drought | 40.37 ± 5.14ᵈ | 14.240±0.786ᵈ | 0.139±0.005ˡ | 0.508±0.015ᵒ | 1.06±0.05^bc^ | 0.21±0.01^b^ | 3.16±0.06ᵉ | 39.80±3.54ᵈ | 254.00±16.73ᵈ | 170.84±10.1ᵉ | 3.42±0.04ᵃ | 0.075±0.001ᵃ |
| *Streptomyces jiujiangensis* NA-06 | Non-stress | 53.17 ± 4.96ᵃᵇᶜ | 65.433±2.650ᵃ | 0.203±0.006ᶜᵈ | 1.182±0.021ᵈᵉ | 1.54±0.02ᵃ | 0.29±0.004^a^ | 2.66±0.06ʰᶦ | 38.43±1.93ᵈ | 172.70±4.35ᶠᵍ | 98.83±2.18ᶠ | 0.87±0.02ʲᵏ | 0.032±0.000ʲ |
|  | Drought | 52.26 ± 3.8ᵃᵇᶜ | 21.500±3.404ᵇᶜᵈ | 0.162±0.006ʰⁱʲᵏ | 0.742±0.021ʲᵏ | 0.85±0.02^de^ | 0.17±0.003ᶜ^d^ | 3.91±0.07ᶜ | 61.37±3.07ᵇᶜ | 331.73±6.62ᵇ | 198.90±6.26ᵇᶜ | 2.88±0.03ᶜ | 0.057±0.000ᵈ |
| *Burkholderia cepacia* NA-07 | Non-stress | 59.19 ± 5.46ᵃᵇ | 67.300±2.452ᵃ | 0.196±0.005ᶜᵈᵉ | 1.156±0.02ᵈᵉᶠ | 1.51±0.03ᵃ | 0.28±0.01^a^ | 2.71±0.06ᵍʰᶦ | 40.13±1.76ᵈ | 215.50±4.95ᵉ | 104.67±2.40ᶠ | 0.91±0.02ⁱʲ | 0.027±0.001ˡ |
|  | Drought | 59.02 ± 2.13ᵃᵇ | 26.600±3.219ᵇ | 0.175±0.006ᶠᵍʰ | 0.791±0.019ⁱʲ | 0.75±0.02^ef^ | 0.15±0.004^de^ | 4.76±0.06ᵃ | 72.87±3.68ᵃ | 406.97±7.87ᵃ | 232.60±5.90ᵃ | 2.20±0.02ᵉ | 0.051±0.000ᵉ |
| *Pantoea dispersa* NA-08 | Non-stress | 55.11 ± 1.7ᵃᵇᶜ | 67.667±0.404ᵃ | 0.213±0.006ᵇᶜ | 1.205±0.019ᶜᵈ | 1.52±0.02ᵃ | 0.28±0.004^a^ | 2.80±0.06ᶠᵍʰ | 40.77±2.01ᵈ | 217.67±4.75ᵉ | 105.73±2.18ᶠ | 0.82±0.02ᵏˡ | 0.028±0.000ᵏˡ |
|  | Drought | 53.04 ± 2.8ᵃᵇᶜ | 26.900±0.400ᵇ | 0.198±0.006ᶜᵈ | 0.868±0.021ʰ | 0.8±0.07^de^ | 0.16±0.02^cd^ | 4.78±0.07ᵃ | 74.10±3.58ᵃ | 409.60±9.10ᵃ | 235.47±6.06ᵃ | 2.19±0.03ᵉ | 0.049±0.001ᶠ |
| *Pseudomonas* sp. NA-15 | Non-stress | 51.38 ± 1.3ᵃᵇᶜ | 62.767±4.162ᵃ | 0.180±0.005ᵉᶠᵍ | 1.13±0.014ᵉᶠᵍ | 1.52±0.04ᵃ | 0.28±0.01^a^ | 2.30±0.05ᵏ | 36.57±2.14ᵈ | 136.23±4.95ʰᶦ | 96.90±2.25ᶠ | 1.04±0.02ᵍʰ | 0.045±0.000ᵍ |
|  | Drought | 46.33 ± 1.34ᶜᵈ | 16.467±2.371ᶜᵈ | 0.146±0.005ᵏˡ | 0.592±0.013ⁿ | 0.59±0.05ᶠ | 0.12±0.01ᵉ | 3.22±0.05ᵉ | 53.10±3.58ᶜ | 265.83±5.72ᵈ | 178.50±6.65ᵈᵉ | 3.43±0.04ᵃ | 0.074±0.001ᵃ |
| *Variovorax guangxiensis* NA-16 | Non-stress | 59.69 ± 4.43ᵃ | 63.167±3.208ᵃ | 0.223±0.007ᵃᵇ | 1.286±0.023ᵃᵇ | 1.59±0.04ᵃ | 0.23±0.01^a^ | 2.84±0.06ᶠᵍ | 37.47±1.99ᵈ | 140.80±5.40ʰᶦ | 97.97±2.27ᶠ | 0.78±0.02ˡᵐ | 0.029±0.000ᵏ |
|  | Drought | 49.33 ± 1.94ᵇᶜᵈ | 16.800±3.676ᶜᵈ | 0.171±0.007ᶠᵍʰᶦ | 0.628±0.02ᵐⁿ | 1.1±0.1^b^ | 0.21±0.02^b^ | 3.25±0.05ᵉ | 52.17±4.54ᶜ | 270.57±5.81ᵈ | 182.90±6.26ᵈᵉ | 3.44±0.04ᵃ | 0.058±0.000ᵈ |
| Consortium-I | Non-stress | 58.46 ± 1.07ᵃᵇ | 66.133±0.351ᵃ | 0.231±0.008ᵃ | 1.324±0.022ᵃ | 1.49±0.02ᵃ | 0.27±0.004^a^ | 2.95±0.07ᶠ | 39.43±2.15ᵈ | 189.07±4.45ᶠ | 101.37±2.24ᶠ | 0.72±0.02ᵐ | 0.032±0.000ʲ |
|  | Drought | 55.69 ± 0.97ᵃᵇᶜ | 23.767±1.350ᵇᶜ | 0.187±0.008ᵈᵉᶠ | 0.812±0.021ⁱ | 0.71±0.04^ef^ | 0.14±0.01^de^ | 4.21±0.07ᵇ | 67.30±3.32ᵃᵇ | 271.23±5.86ᵈ | 212.70±6.20ᵇ | 2.63±0.03ᵈ | 0.052±0.000ᵉ |
| Consortium-II | Non-stress | 55.08 ± 4.54ᵃᵇᶜ | 64.667±2.237ᵃ | 0.188±0.005ᵈᵉᶠ | 1.248±0.018ᵇᶜ | 1.52±0.02ᵃ | 0.28±0.004^a^ | 2.54±0.06ᶦʲ | 37.97±1.89ᵈ | 154.33±4.75ᵍʰ | 99.17±2.24ᶠ | 0.97±0.02ʰᶦ | 0.036±0.000ⁱ |
|  | Drought | 54.55 ± 1.28ᵃᵇᶜ | 19.500±3.600ᵇᶜᵈ | 0.156±0.005ᶦʲᵏˡ | 0.662±0.014ˡᵐ | 0.93±0.03^cd^ | 0.18±0.01ᵇᶜ | 3.56±0.06ᵈ | 59.10±3.16ᵇᶜ | 302.50±6.25ᶜ | 192.03±5.48ᶜᵈ | 3.13±0.03ᵇ | 0.069±0.001ᵇ |
| Consortium-III | Non-stress | 54.00 ± 3.30ᵃᵇᶜ | 64.200±4.729ᵃ | 0.173±0.005ᶠᵍʰ | 1.108±0.015ᶠᵍ | 1.53±0.02ᵃ | 0.29±0.004^a^ | 2.40±0.05ʲᵏ | 38.30±1.85ᵈ | 150.70±5.96ʰ | 99.73±2.32ᶠ | 1.08±0.02ᶠᵍ | 0.040±0.000ʰ |
|  | Drought | 50.65 ± 0.40ᵃᵇᶜ | 19.200±3.341ᵇᶜᵈ | 0.151±0.005ʲᵏˡ | 0.694±0.017ᵏˡ | 0.85±0.14ᵈᵉ | 0.17±0.03ᶜᵈ | 3.58±0.07ᵈ | 59.83±3.16ᵇᶜ | 298.60±6.20ᶜ | 193.73±5.58ᶜᵈ | 3.15±0.03ᵇ | 0.063±0.001ᶜ |

Values are presented as mean ± standard deviation (SD) of three biological replicates (n = 3). Data were analyzed using two-way analysis of variance (ANOVA) to evaluate the effects of bacterial treatments on Ranjit under control vs. drought conditions, and their interaction. Mean comparisons were performed using Tukey’s honestly significant difference (HSD) test. Different lowercase letters indicate statistically significant differences among treatments. Here, SL- shoot length**, RWC- relative water content, RDW- root dry weight, SDW- shoot dry weight, TC- total chlorophyll content, CAR- carotenoid content, TPC- total phenol content, PRO-proline content, SOD-Superoxide dismutase, CAT- catalase activity, H_2_O_2_- hydrogen peroxide content, MDA- malondialdehyde content.**

| **Effects** | **df** | **SL (cm)** | | | | **RWC (%)** | | | | **RDW (g plant⁻¹)** | | | | |
| --- | --- | --- | --- | --- | --- | --- | --- | --- | --- | --- | --- | --- | --- | --- |
|  |  | **Sum Sq.** | **Mean Sq.** | **F value** | **p value** | **Sum Sq.** | **Mean Sq.** | **F value** | **p value** | **Sum Sq.** | **Mean Sq.** | **F value** | **p value** |  |
| Conditions (C) | **1** | 239.7176 | 239.7176 | 22.4102 | < 0.001^***^ | 26735.60 | 26735.60 | 3224.9342 | < 0.001^***^ | 0.01386 | 0.01386 | 422.9650 | < 0.001^***^ |  |
| Treatments (T) | **8** | 699.0953 | 87.3869 | 8.1694 | < 0.001^***^ | 450.9147 | 56.3643 | 6.7988 | < 0.001^***^ | 0.01956 | 0.002445 | 74.6232 | < 0.001^***^ |  |
| C × T | **8** | 238.2750 | 29.7844 | 2.7844 | < 0.05^*^ | 105.0137 | 13.1267 | 1.5834 | 0.1643^ns^ | 0.001783 | 0.000223 | 6.8027 | < 0.001^***^ |  |
| Residuals | **36** | 385.0846 | 10.6968 | — | — | 298.4500 | 8.2903 | — | — | 0.001179 | 3.2759e-5 | — | — |  |
|  | | | | | | | | | | | | | | |
| **Effects** | **df** | **SDW (g plant⁻¹)** | | | | **TC (mg g⁻¹ FW)** | | | | **CAR (mg g⁻¹ FW)** | | | | |
|  |  | **Sum Sq.** | **Mean Sq.** | **F value** | **p value** | **Sum Sq.** | **Mean Sq.** | **F value** | **p value** | **Sum Sq.** | **Mean Sq.** | **F value** | **p value** |  |
| Conditions (C) | **1** | 3.2708 | 3.2708 | 10173.03 | < 0.001^***^ | 6.31 | 6.31 | 2325.83 | < 0.001^***^ | 0.19 | 0.19 | 1734.97 | < 0.001^***^ |  |
| Treatments (T) | **8** | 0.33433 | 0.04179 | 129.9828 | < 0.001^***^ | 0.43 | 0.05 | 19.90 | < 0.001^***^ | 0.02 | 0.002 | 18.93 | < 0.001^***^ |  |
| C × T | **8** | 0.14287 | 0.01786 | 55.5454 | < 0.001^***^ | 0.22 | 0.03 | 10.25 | < 0.001^***^ | 0.009 | 0.001 | 9.93 | < 0.001^***^ |  |
| Residuals | **36** | 0.01157 | 0.000322 | — | — | 0.1 | 0.003 | — | — | 0.004 | 0.0001 | — | — |  |
|  | | | | | | | | | | | | | | |
| **Effects** | **df** | **TPC (mg GAE g⁻¹ FW)** | | | | **PRO (µmol g⁻¹ FW)** | | | | **SOD (U min⁻¹ g⁻¹ FW)** | | | | |
|  |  | **Sum Sq.** | **Mean Sq.** | **F value** | **p value** | **Sum Sq.** | **Mean Sq.** | **F value** | **p value** | **Sum Sq.** | **Mean Sq.** | **F value** | **p value** |  |
| Conditions (C) | **1** | 20.5597 | 20.5597 | 5681.7932 | < 0.001^***^ | 6266.2017 | 6266.2017 | 655.0037 | < 0.001^***^ | 284127.57 | 284127.57 | 5745.0878 | < 0.001^***^ |  |
| Treatments (T) | **8** | 8.3588 | 1.0449 | 288.7524 | < 0.001^***^ | 1787.6326 | 223.4541 | 23.3576 | < 0.001^***^ | 97145.18 | 12143.15 | 245.5356 | < 0.001^***^ |  |
| C × T | **8** | 3.1230 | 0.39038 | 107.8834 | < 0.001^***^ | 1075.7467 | 134.4683 | 14.0559 | < 0.001^***^ | 14054.28 | 1756.7853 | 35.5224 | < 0.001^***^ |  |
| Residuals | **36** | 0.13027 | 0.003619 | — | — | 344.4000 | 9.5667 | — | — | 1780.4067 | 49.4557 | — | — |  |
|  | | | | | | | | | | | | | | |
| **Effects** | **df** | **CAT (U min⁻¹ g⁻¹ FW)** | | | | **H₂O₂ (µmol g⁻¹ FW)** | | | | **MDA (µmol g⁻¹ FW)** | | | | |
|  |  | **Sum Sq.** | **Mean Sq.** | **F value** | **p value** | **Sum Sq.** | **Mean Sq.** | **F value** | **p value** | **Sum Sq.** | **Mean Sq.** | **F value** | **p value** |  |
| Conditions (C) | **1** | 135649.78 | 135649.78 | 5424.6800 | < 0.001^***^ | 54.7828 | 54.7828 | 80827.11 | < 0.001^***^ | 0.009192 | 0.009192 | 36473.41 | < 0.001^***^ |  |
| Treatments (T) | **8** | 8731.0893 | 1091.3862 | 43.6449 | < 0.001^***^ | 4.1459 | 0.51824 | 764.6127 | < 0.001^***^ | 0.003363 | 0.000420 | 1668.2245 | < 0.001^***^ |  |
| C × T | **8** | 4285.4180 | 535.6773 | 21.4219 | < 0.001^***^ | 2.4014 | 0.30017 | 442.8750 | < 0.001^***^ | 0.000235 | 2.9407e-5 | 116.6910 | < 0.001^***^ |  |
| Residuals | **36** | 900.2175 | 25.0060 | — | — | 0.02440 | 0.000678 | — | — | 9.0723e-6 | 2.5201e-7 | — | — |  |

**Supplementary Table 9** Two-way ANOVA F-values and probability (p) for physiological, biochemical, and antioxidant responses of Ranjit with bacterial inoculation under non-stress and drought conditions, with Tukey HSD post-hoc comparisons

Here, *** p < 0.001 (highly significant); ** p < 0.01 (highly significant); * p < 0.05 (significant); ns = not significant

**Supplementary Table 10** Comparative analysis soil enzymatic activities of CR Dhan 802 and Ranjit with bacterial inoculation under non-stress and drought conditions

| **Treatments** | **Conditions** | **CR Dhan 802** | | | **Ranjit** | | |
| --- | --- | --- | --- | --- | --- | --- | --- |
|  |  | **ACP**  **(µg p-nitrophenol g^-1^ dwt h^-1^)** | **DHA**  **(TPF µg^-1^ dwt^-1^ 24h^-1^)** | **URE**  **(µg NH^+^_4_ N g^-1^ dwt 2h^-1^)** | **ACP**  **(µg p-nitrophenol g^-1^ dwt h^-1^)** | **DHA**  **(TPF µg^-1^ dwt^-1^ 24h^-1^)** | **URE**  **(µg NH^+^_4_ N g^-1^ dwt 2h^-1^)** |
| NT-Ck | Non-stress | 249±7^jk^ | 28.37±0.75^jk^ | 56.77±1.55^kl^ | 191.00±6.00^kl^ | 19.23±0.65^lm^ | 43.57±1.45^hi^ |
|  | Drought | 182±6^n^ | 19.50±0.60^m^ | 41.10±1.50^p^ | **123.00±5.00^p^** | 9.67±0.45^p^ | **27.93±1.15^m^** |
| *Streptomyces jiujiangensis* NA-06 | Non-stress | 344.33±7.5^bc^ | 44.53±0.85^c^ | 77.23±1.45^bc^ | 262.67±6.51^cd^ | 31.30±0.70^d^ | 60.60±1.30^cd^ |
|  | Drought | 289.33±7.5^fgh^ | 33.70±0.70^g^ | 61.97±1.55^hij^ | 197.00±6.00^jkl^ | 20.40±0.60^kl^ | 44.20±1.40^hi^ |
| *Burkholderia cepacia* NA-07 | Non-stress | 360.67±8.02^ab^ | 47.50±0.90^b^ | 80.77±1.55^ab^ | 248.33±6.51^de^ | 28.87±0.7^5e^ | 57.13±1.25^de^ |
|  | Drought | 307.33±7.5^de^ | 36.40±0.80^f^ | 65.70±1.60^fgh^ | 227.33±6.51^fgh^ | 25.37±0.65^gh^ | **51.00±1.40^fg^** |
| *Pantoea dispersa* NA-08 | Non-stress | 328.33±7.51^cd^ | 41.73±0.85^d^ | 73.90±1.50^cd^ | 278.00±7.00^bc^ | 34.00±0.80^c^ | 64.10±1.30^bc^ |
|  | Drought | 325.67±8.02^cd^ | 39.23±0.85^e^ | 58.57±1.55^jkl^ | 243.00±7.00^def^ | 28.07±0.75^ef^ | 54.67±1.45^ef^ |
| *Pseudomonas* sp. NA-15 | Non-stress | 296.33±7.51^efg^ | **36.13±0.75^f^** | 67.23±1.45^efg^ | 220.67±6.51^ghi^ | 24.03±0.65^hi^ | 50.57±1.25^fg^ |
|  | Drought | 199.33±6.51^mn^ | 21.63±0.65^m^ | 44.90±1.50^op^ | 139.33±5.51^op^ | 11.60±0.50^p^ | 31.63±1.25^lm^ |
| *Variovorax guangxiensis* NA-16 | Non-stress | 378.33±8.51^a^ | 50.70±0.90^a^ | 84.77±1.65^a^ | 293.33±7.51^ab^ | 36.80±0.80^b^ | 67.77±1.35^ab^ |
|  | Drought | 271.33±7.51^hij^ | 31.20±0.70^h^ | 69.40±1.60^def^ | **154.00±6.00^no^** | 13.77±0.55^o^ | 35.13±1.35^kl^ |
| Consortium-I | Non-stress | 312.33±7.51^de^ | 38.90±0.80^e^ | **70.57±1.45^de^** | 309.67±8.02^a^ | 39.77±0.85^a^ | 71.83±1.60^a^ |
|  | Drought | 253.00±7.00^jk^ | 28.80±0.70^ij^ | 55.10±1.50^lm^ | 212.33±6.51^hij^ | 22.87±0.65^ij^ | 47.57±1.45^gh^ |
| Consortium-II | Non-stress | 265.00±7.00^ij^ | 30.90±0.70^hi^ | 60.30±1.40^ijk^ | 235.00±7.00^efg^ | 26.50±0.70^fg^ | 53.83±1.25^ef^ |
|  | Drought | 217.00±7.00^lm^ | **24.00±0.70^l^** | 48.20±1.50^no^ | 182.33±6.51^lm^ | 18.00±0.60^m^ | **40.77±1.35^ij^** |
| Consortium-III | Non-stress | 281.00±7.00^ghi^ | 33.57±0.75^g^ | 63.87±1.45^ghi^ | 207.33±6.51^ijk^ | 21.80±0.70^jk^ | 47.53±1.35^gh^ |
|  | Drought | 235.00±7.00^kl^ | 26.40±0.70^k^ | 51.67±1.55^mn^ | 168.00±6.00^mn^ | **15.87±0.55^n^** | 37.90±1.30^jk^ |

Values are presented as mean ± standard deviation (SD) of three biological replicates (n = 3). Data were analyzed using two-way analysis of variance (ANOVA) to evaluate the effects of bacterial treatments on CR Dhan 802 and Ranjit under control and drought conditions, and their interaction. Mean comparisons were performed using Tukey’s honestly significant difference (HSD) test. Different lowercase letters indicate statistically significant differences among treatments. Here, **ACP- soil acid phosphatase activity, DHA- soil dehydrogenase activity, and URE-soil urease activity.**

**Supplementary Table 11** Two-way ANOVA F-values and probability (p) for soil enzymatic activities of CR Dhan 802 and Ranjit with bacterial inoculation under non-stress and drought conditions, with Tukey HSD post-hoc comparisons

| **Effects** | **df** | **ACP (µg p-nitrophenol g^-1^ dwt h^-1^)** | | | | **DHA (TPF µg^-1^ dwt^-1^ 24h^-1^)** | | | | **Urease (µg NH^+^_4_ N g^-1^ dwt 2h^-1^)** | | | |
| --- | --- | --- | --- | --- | --- | --- | --- | --- | --- | --- | --- | --- | --- |
|  |  | **Sum Sq.** | **Mean Sq.** | **F value** | **p value** | **Sum Sq.** | **Mean Sq.** | **F value** | **p value** | **Sum Sq.** | **Mean Sq.** | **F value** | **p value** |
| **Variety:**  **CR Dhan 802** | | | | | | | | | | | | | |
| **Conditions (C)** | 1 | 47763.63 | 47763.63 | 888.4726 | < 0.001^***^ | 1394.3585 | 1394.3585 | 2394.1291 | < 0.001^***^ | 3210.9067 | 3210.9067 | 1393.2419 | < 0.001^***^ |
| **Treatments (T)** | 8 | 93682.37 | 11710.30 | 217.8285 | < 0.001^***^ | 2197.5333 | 274.6917 | 471.6486 | < 0.001^***^ | 4241.3100 | 530.1638 | 230.0429 | < 0.001^***^ |
| **C × T** | 8 | 10982.37 | 1372.7963 | 25.5360 | < 0.001^***^ | 281.0215 | 35.1277 | 60.3146 | < 0.001^***^ | 104.1100 | 13.0137 | 5.6468 | < 0.001^***^ |
| **Residuals** | 36 | 1935.3333 | 53.7593 | — | — | 20.9667 | 0.58241 | — | — | 82.9667 | 2.3046 | — | — |
|  | | | | | | | | | | | | | |
| **Variety: Ranjit** | | | | | | | | | | | | | |
| **Conditions (C)** | 1 | 59933.35 | 59933.35 | 1413.2755 | < 0.001^***^ | 1558.4817 | 1558.4817 | 3474.7320 | < 0.001^***^ | 3559.1585 | 3559.1585 | 1957.1747 | < 0.001^***^ |
| **Treatments (T)** | 8 | 62190.70 | 7773.8380 | 183.3132 | < 0.001^***^ | 1659.0633 | 207.3829 | 462.3731 | < 0.001^***^ | 3269.8015 | 408.7252 | 224.7572 | < 0.001^***^ |
| **C × T** | 8 | 15705.15 | 1963.1435 | 46.2925 | < 0.001^***^ | 445.4767 | 55.6846 | 124.1523 | < 0.001^***^ | 814.5148 | 101.8144 | 55.9875 | < 0.001^***^ |
| **Residuals** | 36 | 1526.6667 | 42.4074 | — | — | 16.1467 | 0.44852 | — | — | 65.4667 | 1.8185 | — | — |

Here, *** p < 0.001 (highly significant); ** p < 0.01 (highly significant); * p < 0.05 (significant); ns = not significant

**References**

1. Azarbad, H., Bainard, L. D., Agoussar, A., Tremblay, J., and Yergeau, E. (2022). The response of wheat and its microbiome to contemporary and historical water stress in a field experiment. ISME Commun. 2, 62. doi: 10.1038/s43705-022-00151-2
2. Breidenbach, B., Pump, J., & Dumont, M. G. (2016). Microbial community structure in the rhizosphere of rice plants. Frontiers in microbiology, 6, 1537. <https://doi.org/10.3389/fmicb.2015.01537>
3. Chandra, D., Srivastava, R., Gupta, V. V., Franco, C. M., Paasricha, N., Saifi, S. K., et al. (2019). Field performance of bacterial inoculants to alleviate water stress effects in wheat (Triticum aestivum L.). Plant Soil 441, 261–283. doi: 10.1007/s11104-019-04115-9
4. Chen, C., Xin, K., Liu, H., Cheng, J., Shen, X., Wang, Y., et al. (2017). Pantoea alhagi, a novel endophytic bacterium with ability to improve growth and drought tolerance in wheat. Sci. Rep. 7, 41564. doi: 10.1038/srep41564
5. Chen, L., Dodd, I. C., Theobald, J. C., Belimov, A. A., and Davies, W. J. (2013). The rhizobacterium Variovorax paradoxus 5C-2, containing ACC deaminase, promotes growth and development of Arabidopsis thaliana via an ethylene-dependent pathway. J. Exp. Bot. 64, 1565–1573. doi: 10.1093/jxb/ert031
6. Cherif-Silini, H., Thissera, B., Bouket, A. C., Saadaoui, N., Silini, A., Eshelli, M., et al. (2019). Durum wheat stress tolerance induced by endophyte Pantoea agglomerans with genes contributing to plant functions and secondary metabolite arsenal. Int. J. Mol. Sci.20, 3989. doi: 10.3390/ijms20163989
7. Fang, J., Wei, S., Gao, Y., Zhang, X., Cheng, Y., Wang, J., ... & Lu, Z. (2023). Character variation of root space microbial community composition in the response of drought-tolerant spring wheat to drought stress. Frontiers in Microbiology, 14, 1235708. <https://doi.org/10.3389/fmicb.2023.1235708>
8. Huang, X. F., Zhou, D., Lapsansky, E. R., Reardon, K. F., Guo, J., Andales, M. J., et al. (2017). Mitsuaria sp. and Burkholderia sp. from Arabidopsis rhizosphere enhance drought tolerance in Arabidopsis thaliana and maize (Zea mays L.). Plant Soil 419, 523–539. doi: 10.1007/s11104-017-3360-4
9. Kim, Y. N., Khan, M. A., Kang, S. M., Hamayun, M., and Lee, I. J. (2020). Enhancement of drought-stress tolerance of Brassica oleracea var. italica L. by newly isolated Variovorax sp. YNA59. J. Microbiol. Biotechnol. 30, 1500–1509. doi: 10.4014/jmb.2006.06010
10. Lei, Z., Ding, Y., Xu, W., and Zhang, Y. (2023). Microbial community structure in rice rhizosheaths under drought stress. J. Plant Ecol. 16, rtad012. doi: 10.1093/jpe/rtad012
11. Li, G., Wang, K., Qin, Q., Li, Q., Mo, F., Nangia, V., et al. (2023). Integrated microbiome and metabolomic analysis reveal responses of rhizosphere bacterial communities and root exudate composition to drought and genotype in rice (Oryza sativa L.). Rice 16, 19. doi: 10.1186/s12284-023-00636-1
12. Li, H., Guo, Q., Jing, Y., Liu, Z., Zheng, Z., Sun, Y., et al. (2020). Application of Streptomyces pactum Act12 enhances drought resistance in wheat. J. Plant Growth Regul. 39, 122–132. doi: 10.1007/s00344-019-09968-z
13. Li, J., Liu, H., Wang, J., Macdonald, C. A., Singh, P., Cong, V. T., et al. (2025). Drought induced plant microbiome and metabolic enrichments improve drought resistance. Cell. Host Microbe 33, 882–900.e7. doi: 10.1016/j.chom.2025.05.002
14. Naveed, M., Mitter, B., Reichenauer, T. G., Wieczorek, K., and Sessitsch, A. (2014). Increased drought stress resilience of maize through endophytic colonization by Burkholderia phytofirmans PsJN and Enterobacter sp. FD17. Environ. Exp. Bot. 97, 30–39. doi: 10.1016/j.envexpbot.2013.09.014
15. Nunna, S. A. D., and Balachandar, D. (2024). Rhizobacterial community structure differs between landrace and cultivar of rice under drought conditions. Curr. Microbiol.81, 334. doi: 10.1007/s00284-024-03860-0
16. Pang, F., Solanki, M. K., Xing, Y. X., Dong, D. F., and Wang, Z. (2024). Streptomyces improves sugarcane drought tolerance by enhancing phenylalanine biosynthesis and optimizing the rhizosphere environment. Plant Physiol. Biochem. 217, 109236. doi: 10.1016/j.plaphy.2024.109236
17. Qi, M., Berry, J. C., Veley, K. M., O'Connor, L., Finkel, O. M., Salas-González, I., et al. (2022). Identification of beneficial and detrimental bacteria impacting sorghum responses to drought using multi-scale and multi-system microbiome comparisons. ISME J. 16, 1957–1969. doi: 10.1038/s41396-022-01245-4
18. Sandhya, V. S. K. Z., Ali, S. Z., Grover, M., Reddy, G., and Venkateswarlu, B. (2010). Effect of plant growth promoting Pseudomonas spp. on compatible solutes, antioxidant status and plant growth of maize under drought stress. Plant Growth Regul. 62, 21–30. doi: 10.1007/s10725-010-9479-4
19. Santos-Medellı́n, C., Edwards, J., Liechty, Z., Nguyen, B., and Sundaresan, V. (2017). Drought stress results in a compartment-specific restructuring of the rice root associated microbiomes. mBio 8, e00764-17. doi: 10.1128/mBio.00764-17
20. Santos-Medellı́n, C., Liechty, Z., Edwards, J., Nguyen, B., Huang, B., Weimer, B. C., et al. (2021). Prolonged drought imparts lasting compositional changes to the rice root microbiome. Nat. Plants 7, 1065–1077. doi: 10.1038/s41477-021-00967-1
21. Sarma, R. K., and Saikia, R. (2014). Alleviation of drought stress in mung bean by strain Pseudomonas aeruginosa GGRJ21. Plant Soil 377, 111–126. doi: 10.1007/s11104-013-1981-9
22. Sheibani-Tezerji, R., Rattei, T., Sessitsch, A., Trognitz, F., and Mitter, B. (2015). Transcriptome profiling of the endophyte Burkholderia phytofirmans PsJN indicates sensing of the plant environment and drought stress. mBio 6, e00621-15. doi: 10.1128/mBio.00621-15
23. Sun, C., Wang, R., Tang, G., Cai, S., Shi, H., Liu, F., ... & Xiong, Q. (2023). Integrated 16S and metabolomics revealed the mechanism of drought resistance and nitrogen uptake in rice at the heading stage under different nitrogen levels. Frontiers in Plant Science, 14, 1120584. https://doi.org/10.3389/fpls.2023.1120584
24. Sun, L., Yang, Y., Wang, R., Li, S., Qiu, Y., Lei, P., et al. (2020). Effects of exopolysaccharide derived from Pantoea alhagi NX-11 on drought resistance of rice and its efficient fermentation preparation. Int. J. Biol. Macromol. 162, 946–955. doi: 10.1016/j.ijbiomac.2020.06.199
25. Tallapragada, P., Dikshit, R., and Seshagiri, S. (2016). Influence of Rhizophagus spp. and Burkholderia seminalis on the growth of tomato (Lycopersicon esculatum) and bell pepper (Capsicum annuum) under drought stress. Commun. Soil Sci. Plant Anal. 47, 1975–1984. doi: 10.1080/00103624.2016.1216561
26. Tariq, A., Salman, M., Ashraf, M. A., Bukhari, S. A., and Mustafa, G. (2025). Exploring synergistic effects of Pantoea agglomerans BCH-1 and Bacillus pseudomycoides BCH-3 to enhance maize adaptations under drought condition. J. Soil Sci. Plant Nutr. 25,4747–4766. doi: 10.1007/s42729-025-02426-8
27. Tiwari, S., Lata, C., Chauhan, P. S., and Nautiyal, C. S. (2016). Pseudomonas putida attunes morphophysiological, biochemical and molecular responses in Cicer arietinum L. during drought stress and recovery. Plant Physiol. Biochem. PPB 99, 108–117. doi: 10.1016/j.plaphy.2015.11.001
28. Villafañe, D. L., Maldonado, R. A., Bianchi, J. S., Kurth, D., Gramajo, H., Chiesa, M. A., et al. (2024). Streptomyces N2A, an endophytic actinobacteria that promotes soybean growth and increases yield and seed quality under field conditions. Plant Sci. 343,112073. doi: 10.1016/j.plantsci.2024.112073
29. Wang, P., Marsh, E. L., Kruger, G., Lorenz, A., & Schachtman, D. P. (2020). Belowground microbial communities respond to water deficit and are shaped by decades of maize hybrid breeding. *Environmental microbiology*, *22*(3), 889-904. <https://doi.org/10.1111/1462-2920.14701>
30. Wu, C., Zhang, X., Liu, Y., Tang, X., Li, Y., Sun, T., et al. (2024). Drought stress increases the complexity of the bacterial network in the rhizosphere and endosphere of rice (Oryza sativa L.). Agronomy 14, 1662. doi: 10.3390/agronomy14081662
31. Xie, J., Dawwam, G. E., Sehim, A. E., Li, X., Wu, J., Chen, S., et al. (2021). Drought stress triggers shifts in the root microbial community and alters functional categories in the microbial gene pool. Front. Microbiol. 12, 744897. doi: 10.3389/fmicb.2021.744897
32. Xu, F., Liao, H., Zhang, Y., Yao, M., Liu, J., Sun, L., et al. (2022). Coordination of root auxin with the fungus Piriformospora indica and bacterium Bacillus cereus enhances rice rhizosheath formation under soil drying. ISME J. 16, 801–811. doi: 10.1038/s41396-021-01133-3
33. Xu, L., Naylor, D., Dong, Z., Simmons, T., Pierroz, G., Hixson, K. K., et al. (2018). Drought delays development of the sorghum root microbiome and enriches for monoderm bacteria. PNAS 115, E4284–E4293. doi: 10.1073/pnas.1717308115
34. Yasmin, H., Bano, A., Wilson, N. L., Nosheen, A., Naz, R., Hassan, M. N., et al. (2022). Drought-tolerant Pseudomonas sp. showed differential expression of stress-responsive genes and induced drought tolerance in Arabidopsis thaliana. Physiol. Plant 174,e13497. doi: 10.1111/ppl.13497
35. Zhu, J., Li, A., Zhang, J., Sun, C., Tang, G., Chen, L., et al. (2022). Effects of nitrogen application after abrupt drought-flood alternation on rice root nitrogen uptake and rhizosphere soil microbial diversity. Environ. Exp. Bot. 201, 105007. doi: 10.1016/j.envexpbot.2022.105007
